# Speeding up taxonomy in the digital age: A deep learning approach for identifying cryptic freshwater snails

**DOI:** 10.1371/journal.pcbi.1014733

**Published:** 2026-09-02

**Authors:** Dennis Vetter, Muhammad Ahsan, Diana Delicado, Thomas A. Neubauer, Thomas Wilke, Gemma Roig

**Affiliations:** 1 Department of Computer Science, Goethe University Frankfurt, Frankfurt am Main, Germany; 2 The Hessian Center for Artificial Intelligence (hessian.AI), Darmstadt, Germany; 3 Museo Nacional de Ciencias Naturales CSIC, Madrid, Spain; 4 SNSB – Bavarian State Collection for Palaeontology and Geology, Munich, Germany; 5 Department of Earth and Environmental Sciences, Palaeontology & Geobiology, Ludwig-Maximilians-Universität München, Munich, Germany; 6 Department of Animal Ecology & Systematics, Justus Liebig University, Giessen, Germany; 7 Branch for Bioresources, Fraunhofer Institute for Molecular Biology and Applied Ecology IME, Giessen, Germany; University of Minho, PORTUGAL

## Abstract

Cryptic species complexes pose fundamental challenges to biologists, as species exhibit minimal morphological differences that require integrating morphology, genetics, and biogeography for identification. Here, we present a deep learning approach to support species identification in the freshwater snail genus *Radomaniola* (Hydrobiidae), a morphologically cryptic group from the Balkans. Our approach mirrors the integrative workflow of expert taxonomists by combining shell images, morphometric measurements, and collection‑site metadata, with optional phylogenetic information. Despite being trained on fewer than 700 specimens across 20 visually similar species with strongly imbalanced class sizes, the system achieved high identification performance. Careful control of spurious correlations, such as those arising from site‑specific imaging conditions or overly precise geographic metadata, was essential to ensure that the network learned biologically meaningful features. Across all experiments, integrating multiple data types and jointly optimizing meaningful embeddings and classification consistently improved performance over image‑only and classification‑only baselines. On specimens from collection sites seen during training we achieved a macro-averaged F1 score of 0.93. Even though this dropped as low as 0.14 when evaluating on specimens from previously unsampled localities, it could be rapidly recovered by retraining with 2–3 newly labeled specimens. Additionally, model top-3 accuracy stayed consistently above 80% in all settings. These results show that relatively lightweight deep learning models can provide practical decision support in real taxonomic workflows.

## Author summary

Taxonomy, the identification and classification of species, is a time‑consuming task that relies heavily on scarce expert knowledge. In this study, we use Siamese neural networks to support the classification of freshwater snails of the genus *Radomaniola*, a group in which species are notoriously difficult to distinguish based on shell morphology alone. Unlike many machine learning applications that depend on thousands of labeled examples, our system learns effectively from fewer than 700 specimens distributed across 20 visually similar species with very uneven sample sizes. This is achieved by combining high‑quality shell photographs with morphometric measurements and collection‑site information, and by explicitly reducing confounding factors such as site‑specific backgrounds and imaging conditions. We further test the system in realistic scenarios, including cases where specimens originate from previously unsampled sites and where expert labels become available only gradually. These experiments show that the method can provide useful candidate species suggestions and quickly improve as new labeled specimens are added. Overall, our work illustrates how deep learning can be integrated into taxonomic workflows as a practical aid, while leaving final decisions and species delimitation to human experts.

## Introduction

Reliable species identification is a fundamental requirement for taxonomic research (i.e., classification of organisms) and a critical first step in many areas of biology, including conservation, ecology, and evolutionary studies. Key tasks in these fields, such as mapping species distributions, estimating divergence times, monitoring population sizes, or understanding ecological preferences, depend directly on accurate identifications. At the same time, the demand for reliable species-level data is rapidly increasing due to global challenges such as biodiversity loss [1], climate-driven range shifts [2,3], and large-scale ecological monitoring efforts [4]. In parallel, the mass digitization of natural history collections has produced vast image-based datasets [5–7], many of whose specimens remain unidentified or only coarsely and sometimes incorrectly labeled [8]. However, the number of trained taxonomic experts is limited and, in many groups, declining [6,9,10]. This growing imbalance between the demand for identification and the available expertise highlights the need for scalable, automated approaches to species recognition.

Species classification and identification often require years of training and specialization within a particular taxonomic group. Such expertise is unevenly distributed across the tree of life and is especially scarce for inconspicuous or morphologically simple organisms, which nevertheless represent a large proportion of biodiversity [11,12]. The problem is further compounded in taxonomic groups that contain complexes of visually similar or cryptic species, where interspecific differences are subtle and difficult to interpret, even for trained specialists [13]. In these contexts, machine learning approaches have the potential to accelerate identification and support taxonomic workflows by providing preliminary classifications for both experts and non-specialists [14–23]. However, most existing systems rely on large, balanced, and standardized image datasets, conditions that are rarely met in real taxonomic studies. Instead, many datasets are small, imbalanced, and geographically structured, with substantial intraspecific variation across populations [6,16–19]. Under these circumstances, relying on a single data modality may be insufficient. Integrating multiple sources of information, such as images, morphometric measurements, and contextual metadata, offers a promising strategy to capture complementary signals and improve generalization in realistic taxonomic scenarios.

**Fig 1:**
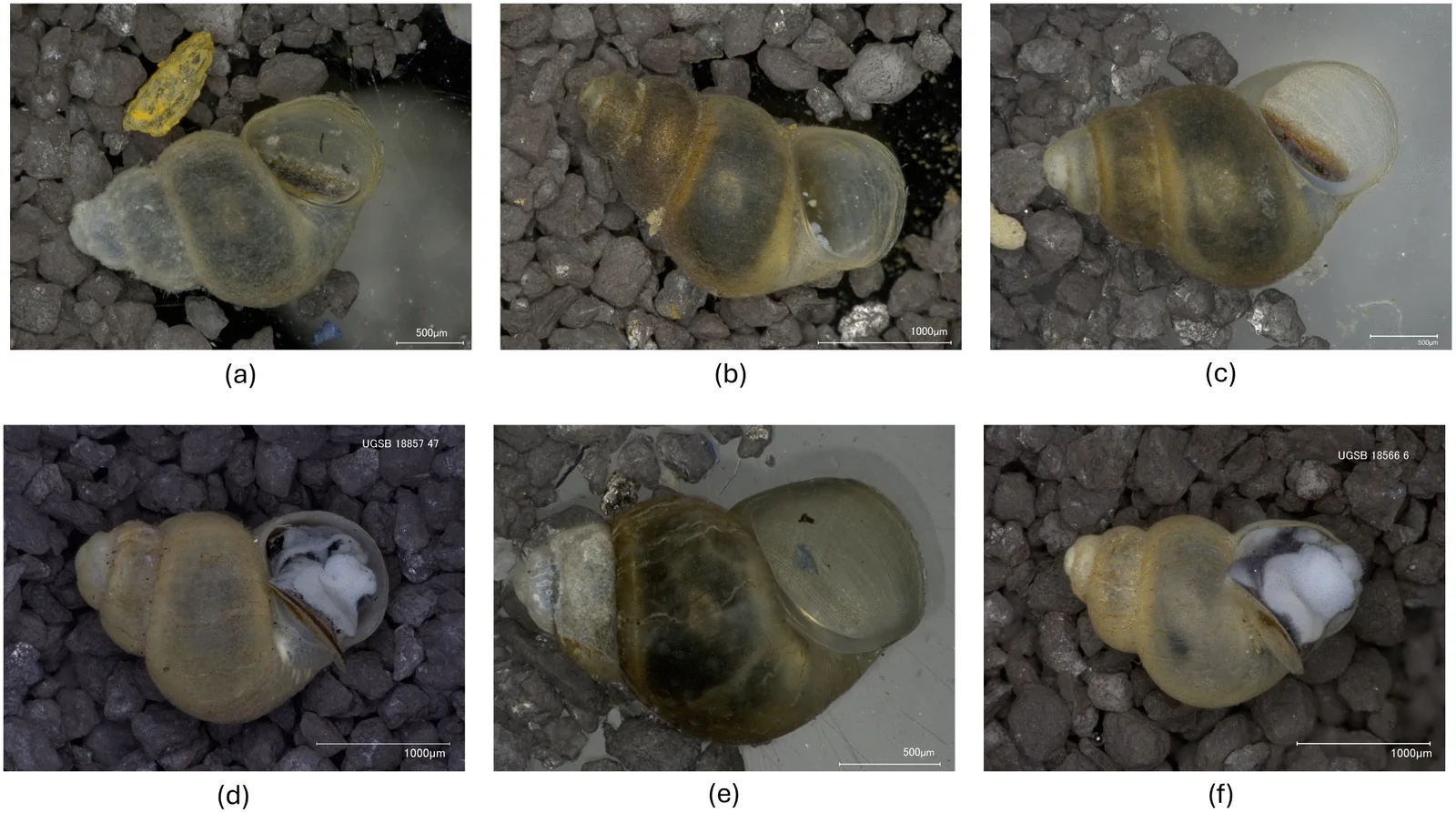
Shells of six cryptic *Radomaniola* species. a: *R. curta,* b: *R. jovanovskae,* c: *R. mostarensis*, d: *R. nachtigallae,* e: *R. seminula,* f: *R. szarowskae*.

Here, we address this challenge using the freshwater snail genus *Radomaniola* Szarowska, 2007 as a model system. This genus exemplifies the challenges of cryptic species identification: its members are morphologically similar gastropods (2–4 mm shell length) inhabiting springs and flowing waters primarily in the Balkan region [24–26], with species delimitation relying primarily on genetic and subtle anatomical characters [25]. As illustrated in Fig 1, morphological differences between species are minimal even for trained taxonomists.

Current taxonomic practice requires multiple, labor-intensive steps, including transferring specimens to a laboratory, detailed examination under a binocular microscope, specimen dissection, DNA extraction, and meticulous comparison with other species within the genus. Additionally, the limited number of experts and their lengthy training process can further delay species identification.

Building a machine learning system to support this taxonomic workflow poses several challenges. First, deep learning systems typically require large amounts of data to perform well. Our dataset of ca. 700 specimens was collected over 18 years [25], reflecting the realistic constraints of taxonomic fieldwork, such as remote collection localities, difficulties related to sampling endangered or rare species, and problems with targeted collection because species identity is often unknown prior to collection and analysis. This results in severely imbalanced class representation.

Second, specimens collected across different sites and years introduce potential confounding variables (e.g., heterogeneous imaging conditions, background variation) that could be exploited as “shortcuts” [27] by machine learning systems rather than learning biologically meaningful features.

Third, taxonomic data are inherently specimen-centered rather than species-centered. Intraspecific variation across populations may approach or exceed interspecific variation, particularly in widespread or geographically structured species [6,16–19].

To address these challenges, we developed a multimodal deep learning system that mirrors the integrative approach of taxonomists by combining shell images, morphometric measurements, and genetic information. Our system is based on Siamese networks, which have shown promise for learning from small, imbalanced biological datasets [22,23]. We demonstrate that this approach achieves high classification accuracy despite severe data limitations, and we evaluate performance under realistic scenarios including classification of specimens from previously unsampled localities and incremental learning as new specimens are identified. Our work demonstrates that computational approaches can effectively support taxonomic workflows while preserving the essential role of expert knowledge, potentially helping to address the “taxonomic impediment” [6] by making taxonomic expertise more accessible and efficient.

## Dataset

**Fig 2:**
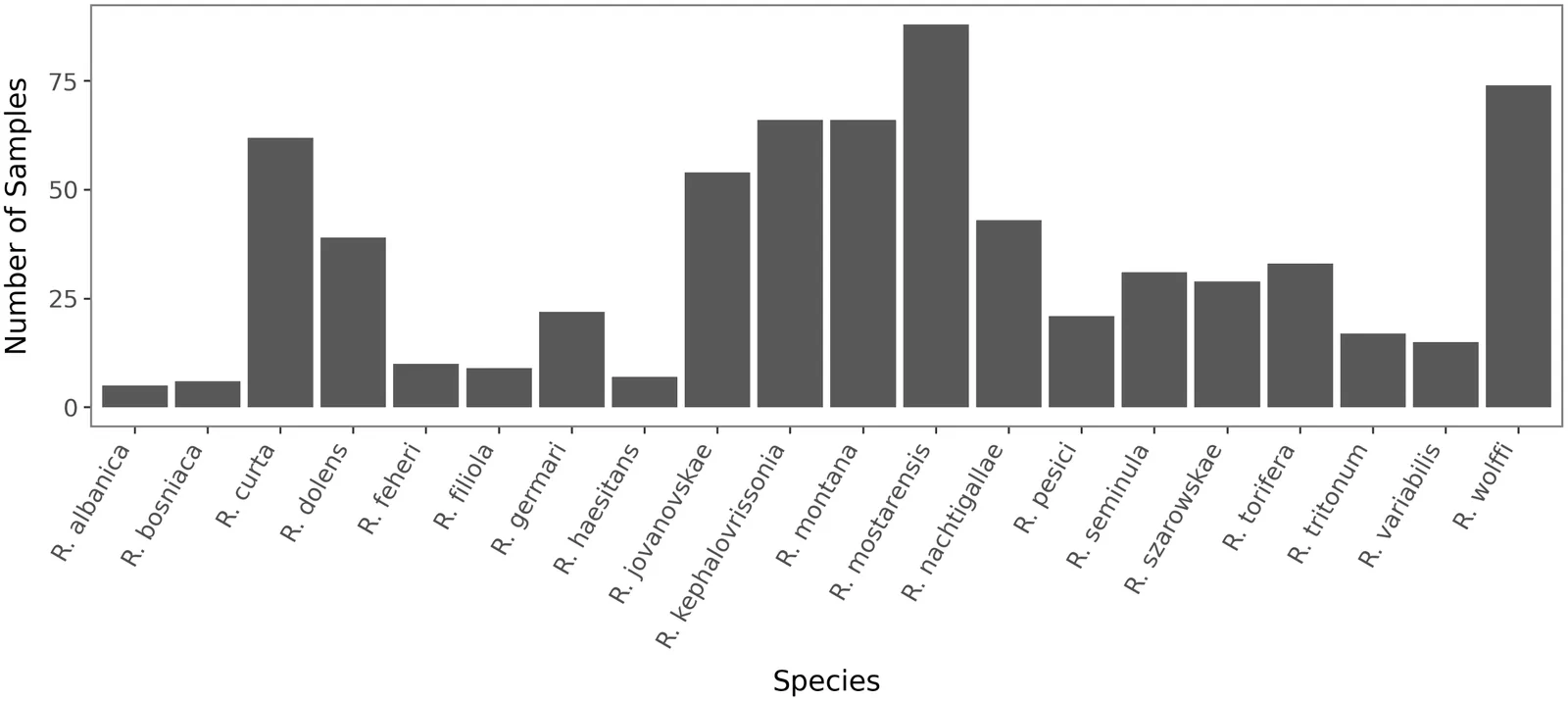
Class imbalance in the *Radomaniola* dataset. Number of specimens per species ranges from 5 (*R. albanica*) to 88 (*R. mostarensis*), reflecting realistic constraints of taxonomic fieldwork.

The dataset used in our work is a comprehensive set of *Radomaniola* specimens, collected over multiple years and classified by domain experts. It consists of 697 specimens distributed across 20 species and 31 collection sites. For each species, between 5 and 88 specimens are available, occurring at 1–5 distinct collection sites. At each collection site, only a single species is encountered. As illustrated in Fig 2, the dataset is severely imbalanced, with multiple species (classes) represented by fewer than 10 specimens.

For each specimen we have a high-resolution photograph available (see Fig 1), in combination with a series of morphometric measurements and metadata from the collection site. All images were taken with the same camera system, a Keyence VHX 2000 digital microscope in combination with the VHX-2000 communication software v. 2.3.5.0 (Keyence Corporation). Not all specimens were preserved; for dissected individuals the shell had to be broken to allow examination of the soft parts. For the remaining specimens, the shell (and the specimen) is preserved and deposited in the Justus Liebig University Giessen (UGSB) collection. All specimens were photographed aperture side up. However, their orientation, distance to the objective, relative size in the image, and spatial resolution (µm per pixel) differ between images; even though relative size in the image and spatial resolution are relatively consistent across photographs from the same collection site. The specimen-specific measurements consist of shell length, shell width, aperture length, aperture width, width of body whorl, and width of penultimate whorl. These variables are standard morphometric characters commonly used in species delimitation and taxonomic studies of Hydrobiidae [28,29]. Collection site metadata consists of country, geographic coordinates, habitat type following IUCN standards [30], local temperature and precipitation from the WorldClim database [31], geological information from The Geological Map of Europe [32], and elevation; all assigned based on collection site coordinates. Climate and geological data were extracted using R v4.3.2 [33], with the packages terra v1.7-55 [34] and rnaturalearth v0.3.4 [35]. The cophenetic distances used in this study were derived from [25], presenting a species classification tree based on Bayesian coalescence. We transformed this phylogenetic clustering into a pairwise distance matrix that indicates similarity between species in time units. Lower distances indicate greater genetic similarities between two species.

Genetic data were not obtained for every individual specimen. Typically, between one and three specimens per population were DNA-sequenced. However, *Radomaniola* species rarely co-occur at the same locality. In addition, approximately half of the specimens were dissected and their species identity was independently confirmed using anatomical characters. Therefore, species assignments are considered reliable despite the lack of genetic data for every specimen.

### Spurious correlation analysis

We analyzed the dataset for potential spurious correlations to ensure that our system focuses on biologically meaningful features rather than artifactual shortcuts. We identified an issue with the specificity of the site-related geographic coordinates and temperature data, which allowed unique identification of collection sites. Because each collection site contained only a single species, models could overfit on site-specific features and simply learn which species was encountered at which location instead of learning generalizable features relevant to the taxonomy task. We verified this issue by training a random forest classifier to predict species using (i) all features, (ii) only geographic coordinates, temperature information, elevation, and precipitation. In both cases, this simple classifier could predict the correct species with 100% accuracy. However, when these site-specific features were removed, the accuracy dropped to slightly above 80%, confirming that these variables enabled shortcut learning rather than contributing to genuine classification ability.

It is important to emphasize that the excluded variables are not necessarily unimportant for species classification. For taxonomists, geographic location and environmental parameters are important descriptors of species habitats. However, in their current form (precise coordinates uniquely identifying sites), these features might be unsuitable for machine learning because they prevent the model from generalizing to new, unsampled locations. We therefore retained only country-level geographic information, which provides coarse biogeographic context without enabling memorization of specific collection sites. For *Radomaniola*, country correlates with species composition but multiple species may occur within the same country, thus preserving biological signal while avoiding overfitting. The full set of features used, together with their value ranges and how they were pre-processed, is included in Table 1.

**Table 1:** Shell measurements and collection site metadata with values and preprocessing.

Feature	Values	Preprocessing
Aperture length [mm]	0.60-1.53 (1.00 ± 0.15)	BatchNorm
Aperture width [mm]	0.22-1.30 (0.82 ± 0.13)	BatchNorm
Shell length [mm]	1.30-3.33 (2.09 ± 0.44)	BatchNorm
Shell width [mm]	0.81-2.10 (1.42 ± 0.23)	BatchNorm
Width of body whorl [mm]	0.70-2.00 (1.26 ± 0.22)	BatchNorm
Width of penultimate whorl [mm]	0.41-1.36 (0.79 ± 0.19)	BatchNorm
Country of collection	‘Albania’, ‘Bosnia and Herz.’, ‘Croatia’, ‘Greece’, ‘Montenegro’, or ‘North Macedonia’	One-hot encoding
ecoregion code (FEOW)	418, 419, 420, 421, or 424	One-hot encoding
Geological information	Three flags: geol_carb, geol_silic, geol_volc	None

Numerical features are listed with value range, mean, and standard deviation. Categorical features are listed with the set of possible values. Numerical features were scaled with a learnable BatchNorm layer, categorical features were one-hot encoded.

### Image and feature preprocessing

**Fig 3:**
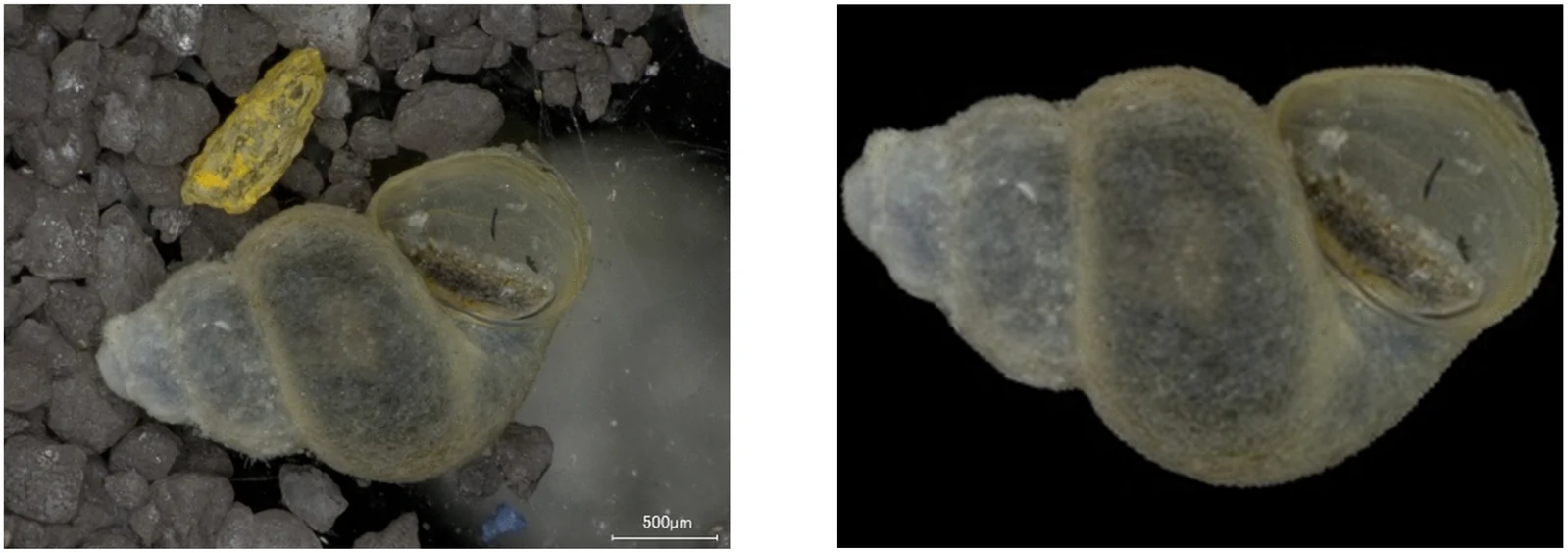
Example of *Radomaniola* shell-image processing. Left: original image with natural background, right: processed image with removed background, standardized orientation, and cropped to size.

The goal of image and feature preprocessing was to minimize spurious correlations from the dataset and enable our system to learn biologically meaningful features. Based on the results of our spurious correlation analysis, we removed all collection site metadata that allowed identification of a specific site, such as precise geographic coordinates, temperature, elevation, and precipitation. The retained features include country, habitat type, geological information, and shell-specific measurements. Furthermore, we processed images to remove information that might enable collection site identification. Specifically, we removed image backgrounds, unified the snails’ relative size, and normalized orientation to ensure the model focuses on visual features of the shell, rather than relying on differences in scaling or orientation. This standardization is particularly important for shell size, where the model should rely on the precise morphometric measurements rather than apparent size in photographs. Image processing was performed with custom software based on Meta’s Segment Anything Model 2 [36]. An example of the original and the processed image is shown in Fig 3.

## Materials and methods

Our system integrates images, shell measurements, geographic features from collection site metadata, and genetic information. We use these multimodal inputs to simultaneously learn an intermediate representation that captures specimen similarities and how to optimize classification performance. In the following, we therefore briefly cover the fundamentals of multi-task learning, multimodal learning, and similarity learning, before presenting the architecture of our final system, and implementation details.

### Transfer learning and multi-task learning

Training deep learning systems on small datasets can easily result in poor performance and overfitting. Two effective techniques to mitigate these issues are transfer learning and multi-task learning. In transfer learning, also known as fine-tuning, the network is first trained on a different, generally much larger dataset. The idea is that during this pre-training phase, the network learns expressive intermediate features that can be leveraged to achieve high performance on similar tasks [37,38].

Multi-task learning aims to enhance performance by exploiting knowledge from related tasks, for example by learning multiple tasks simultaneously while using a shared intermediate representation. This approach has been shown to improve generalization and model performance compared to models trained for the tasks in isolation [39–41], and it can also help alleviate limitations associated with small datasets [42]. A key challenge in multi-task learning is combining the multiple objectives into a single loss function. Loss functions often vary in scale, making the naive approach of weighted combinations difficult to tune. In addition, the value ranges and the magnitude of the losses can change during training. The approach proposed by Kendall et al. [41] addresses this by learning optimal loss weights during training based on the observed variance of the individual loss functions.

In our system, we use a convolutional neural network (CNN) pre-trained on more than 1 million images across 1000 classes of the ImageNet dataset [43] to extract expressive image features. This helps us overcome the limitations of our small dataset and provides a solid foundation for capturing meaningful image features. To address differences in loss scales between embedding- and classification loss, we adopt the method proposed in [41] to dynamically learn appropriate loss weights during training.

### Multimodal learning

In multimodal learning, the system learns from multiple input modalities simultaneously [44,45]. Instead of relying only on tabular data, text, or images, the system integrates and learns a combination of these modalities. The rationale is that each modality captures different aspects of the data, and learning from multiple modalities can potentially lead to improved performance [45]. The key challenge in multimodal deep learning is how to efficiently represent the different modalities. A common solution involves creating a separate set of layers for each modality to compute intermediate representations, which are then projected into a joint space. This joint representation is then used for downstream tasks, such as classification [45,46]. Previous research has shown that this approach allows for the entire network to be trained end-to-end to simultaneously learn efficient representations of the different modalities and perform the task at hand [44,47–50].

In this work, we have four modalities of data available: images, shell measurements, collection site metadata, and genetic information. These modalities were identified by domain experts as central to the taxonomic classification process. Images capture structural shell details, genetic data inform about the relationships between species, and shell measurements and collection site metadata provide complementary attributes that are difficult to learn from images alone. By integrating these complementary features through multimodal learning, our system improves decision accuracy.

### Similarity learning

A technical approach to alleviating the limitations of small datasets is to focus on learning a similarity metric on input data rather than directly training a classifier. This includes embedding the inputs, that is mapping the inputs to high-dimensional real vectors such that the easy-to-compute similarity between these vectors reflects the more difficult-to-formalize similarity between the inputs [22,23,51–54].

One approach to learning this mapping is through Siamese networks [54,55]. Siamese networks are trained on pairs of inputs to produce small distances between the embeddings of inputs from the same class and large distances between embeddings of the inputs from different classes [54,55]. This approach has been shown to work well with the comparatively small datasets used in biological applications, where the datasets often have many classes and few samples per class. Examples include the classification of snakes [23] and plants [22].

An extension of Siamese networks are triplet networks [52], which are trained not on pairs, but on triplets of inputs: an anchor, a positive example from the same class as the anchor, and a negative example from a different class. The training goal is to produce small distances between the embeddings of the anchor and the positive example and larger distances between the embeddings of the anchor and the negative example. This can produce embeddings that capture the similarities in the underlying dataset in a way that is better suited for classification tasks [52,56]. In addition, these embeddings have been shown to work well with imbalanced datasets and with only a few samples per class [56]. A drawback of triplet networks is their sensitivity to the selection of triplet candidates [56–58]. Extensions have been proposed that replace the positive and negative examples with learnable class centers [58,59], or replace the fixed margin with a learnable class-dependent margin [60]. For a comprehensive review of embedding techniques, we refer to [61]. In their benchmark comparisons, the authors showed that there is no single “best” method for embedding learning, performance depends on dataset specifics. They also showed that Siamese networks and triplet networks form a strong baseline over which often only marginal improvements are possible.

Siamese networks, triplet networks, and their extensions are supervised methods that rely on class labels to optimize intra- and inter-class similarities. A different approach are self-supervised learning (SSL) methods that do not rely on the availability of class labels and instead work with unlabeled data [62–66]. Instead of finding positive and/ or negative samples based on class labels, these methods use augmentations to create different “views” of the same input sample. Then, the similarity between views from the same sample is increased, and the similarity between views from different samples is decreased. This allows learning on large datasets without reliance on human annotators [67], which, especially in taxonomy, are often a bottleneck. At the same time, it was demonstrated that SSL methods can produce representations that allow classification performance at the same level as fully supervised methods [62–66]. These methods can be of special relevance for taxonomic approaches, as they also allow learning from large collections of specimens that did not go through the time-consuming manual taxonomic process yet. SSL methods can also be used in combination with supervised methods to allow learning from large batches of unlabeled data, while also including potentially available class labels to improve the representations [68–70].

In our work, we leverage similarity learning approaches to produce meaningful representations that help us address the challenges posed by our small, imbalanced dataset, for which many classes are represented only by a small number of samples. In addition, we leverage a combination of supervised and self-supervised learning to learn from both unlabeled data and the available labeled data.

### System description

**Fig 4:**
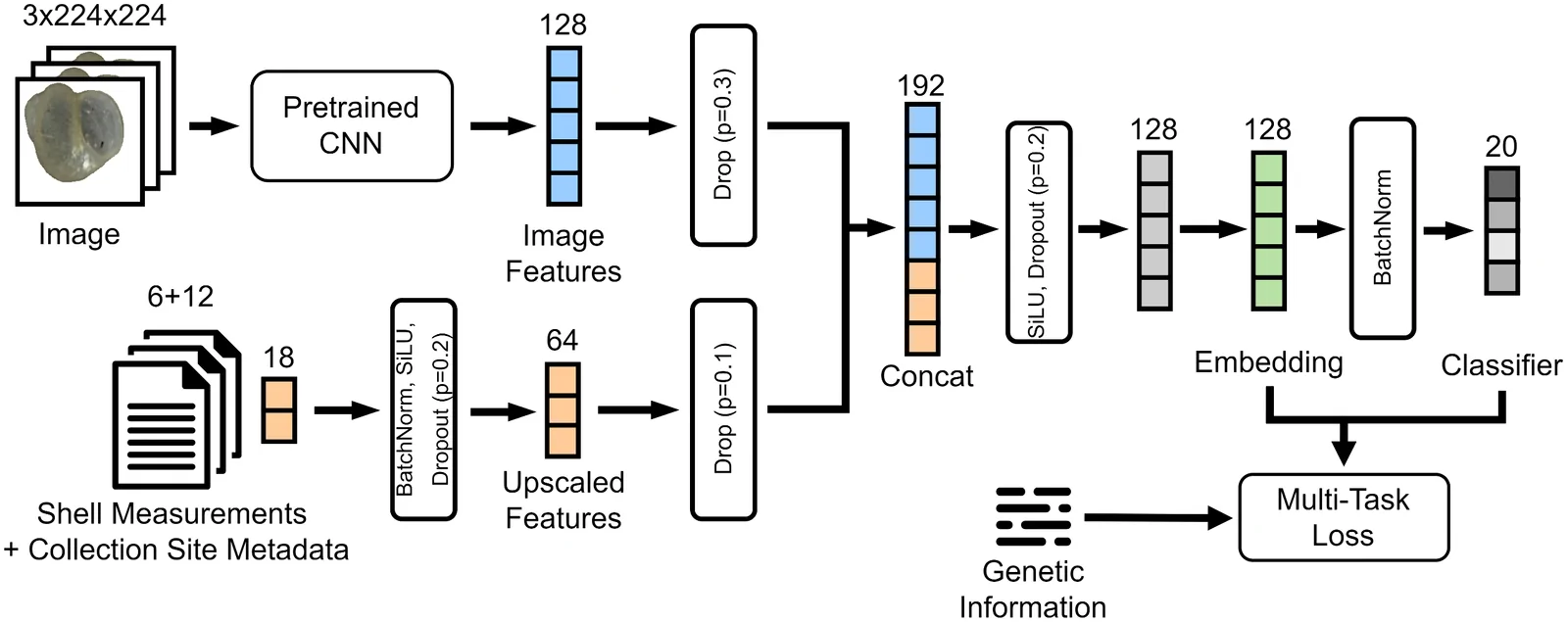
Architecture of the classification system. A pre-trained CNN extracts image features from the input images. The image features and the upscaled measurements are concatenated to create a joint representation, which is mapped to an embedding used for classification. Genetic information is incorporated only during training to inform the joint optimization of embeddings and classification results.

The goal of our system is to learn a robust classification of the small and severely imbalanced *Radomaniola* dataset by integrating data from multiple modalities. As illustrated in Fig 4, our system consists of the following steps:

- Image feature extraction,
- Integrating image features, shell measurement, and collection site metadata into a joint representation,
- Learning embeddings from the joint representation,
- Using embeddings for classification.

The backbone of our system is a MobileNetV3-small network [71], pre-trained on the ImageNet dataset [43], which maps input images to 128-dimensional image feature vectors. During training we only optimize the last layers, namely convolutional block 5, conv head, and the classifier layer that outputs the 128-dimensional image feature vector. All other parameters, including convolutional blocks 0-4 remain fixed to their pre-trained values. To better balance the number of measurements with the number of image features, we upscale the measurements to 64 with a single fully-connected layer. The upscaled measurements are then integrated into a joint representation through concatenation with the image feature vector, as illustrated by the Concat layer in Fig 4. This joint representation is then mapped to a 128-dimensional embedding with two fully-connected layers. The embedding is optimized using the contrastive loss [53,72] commonly used in Siamese networks [22,23,51]. For inputs *x*_*1*_*, x*_*2*_ and margin *m*, the contrastive loss is given as:


$$\begin{array}{c} \begin{array}{c} L_{e}\left(x_{\text{1}},x_{\text{2}},Y\right)=Y\cdot d\left(x_{\text{1}},x_{\text{2}}\right)+(\text{1}-Y)\cdot \text{max}\left(\text{0},\;m-d\left(x_{\text{1}},x_{\text{2}}\right)\right)\; \end{array} \end{array}$$ (1)

Here, *d(x*_*1*_*, x*_*2*_*)* is the distance in embedding space, and *Y* = 1 if the samples *x*_*1*_*, x*_*2*_ are from the same class, and *Y* = 0 otherwise. Optimizing this loss function reduces the distance between the embeddings of samples from the same class and increases the distance between embeddings from different classes. Following [73] we L2-normalize embeddings for computation of the embedding loss, but use unnormalized embeddings followed by a BatchNorm layer as input for the classifier. In addition, both image features and upscaled measurement features are randomly dropped (i.e., set to zero) to ensure the model is not only focusing on one modality.

We also aim for biologically meaningful embeddings that capture the similarities between species. To achieve this, we replaced the fixed margin *m* in Equation (1) with a dynamic margin *m(x*_*1*_*, x*_*2*_*)*. The dynamic margin depends on the classes of the samples, and instead of a fixed value, it returns a margin proportional to the intrinsic differences between the classes. In our system, we used a matrix of genetic distances between species, informed by [25], to capture these intrinsic differences between classes. Examples, from our matrix of cophenetic distances include *R. bosniaca* and *R. nachtigallae*, which have a distance of 2.02, and *R. bosniaca* and *R. wolffi*, with a distance of 6.37. The margin between the classes is then a base margin multiplied by the cophenetic distance. Therefore, the margin between *R. bosniaca* and *R. wolffi* is approximately three times as large as the margin between *R. bosniaca* and *R. nachtigallae.* This is comparable to the approach presented in [60], but instead of one learnable margin per sample class, we have a fixed margin for each pair of classes. Finally, the embeddings are fed into a classification head, where the loss for input x with true label *y,* the classification loss *L*_*c*_*(x,y)* is given by the standard cross-entropy loss for classification tasks.

In addition to supervised embedding learning, Siamese networks can also learn under self-supervision. This is commonly achieved with the InfoNCE (sometimes also called NT-Xent) objective [62,63,65,66,68–70] initially introduced by Oord et al. in [64]. For a sample *x_i_* two views and their embeddings *z_i_* and z_i_* are created. The loss for this sample is then defined as:


$$\begin{array}{c} \begin{array}{c} L_{e}\left(x_{i}\right)=-\text{log}\frac{\text{exp}\left(\frac{sim\left(z_{i},\;z_{i}^{\prime }\right)}{\tau }\right)}{\sum_{\text{k}\neq \text{i}}\text{exp}\left(\frac{sim\left(z_{i},\;z_{k}\right)}{\tau }\right)}\; \end{array} \end{array}$$ (2)

Here, *z_k_* are the embeddings of the other samples in the batch, sim is a similarity metric, such as cosine similarity, and τ is a regularization parameter. In contrast to supervised Siamese networks, the self-supervised Siamese network does not rely on class information. Instead, the network reduces the difference in embeddings from different views of the same image, and increases the difference in embeddings from different images. Importantly, this objective does not rely on availability of labels, and therefore also allows inclusion of unlabeled test data during training or other available collections of unlabeled data.

A common approach in embedding learning is to first learn embeddings and then either use these embeddings directly for classification or train a classifier on the embeddings. In our system, we train the complete network end-to-end, optimizing for both embeddings and classification simultaneously. We used the approach described in [41] to automatically learn appropriate normalization factors for each loss during training. The final loss function for our system is given by the following equation, where embedding loss *L*_*e*_, classification loss *L*_*c*_ and their respective weights *(s*_*e*_*, s*_*c*_*)* are jointly optimized:


$$\begin{array}{c} \begin{array}{c} L(x,\;y)=\text{exp}\left(-s_{e}\right)\cdot L_{e}(x,\;y)+\;\text{2}\cdot \text{exp}\left(-s_{c}\right)\cdot L_{c}(x,\;y)+s_{e}+s_{c} \end{array} \end{array}$$ (3)

Here, the embedding loss of a batch *{(x*_*i*_*, y*_*i*_*)}* is computed as the sum of the individual contrastive losses over all pairs of samples in the batch. This combined loss allows us to leverage the ability of Siamese networks and multi-task learning to learn meaningful representations from small sample sizes and imbalanced datasets. At the same time, the weighted joint training ensures that the learned representations are well suited for classification and trains the classifier to optimally use them for the classification task. Finally, the lightweight MobileNet architecture allows for fast inference and training even on systems with limited computational resources.

### Implementation details

All experiments were implemented in PyTorch [41]. The implementation of embedding learning is based on the pytorch metric learning package [42]. Networks were trained for 20 epochs using a batch size of 64 and the Adam optimization method with decoupled weight decay [43]. As optimizer parameters we used a learning rate of 0*.*001, betas of 0*.*9 and 0*.*999 and weight decay factor of 0*.*1. The learning rate is decayed with a cosine schedule to 10^-8^ [74].

Class imbalance was addressed by oversampling during training. Each sample was repeated *k*_*i*_ times, so that $k_{i}\cdot n_{i}\leq n_{max}<\left(k_{i}+\text{1}\right)\cdot n_{i}$ where *n*_*i*_ is the number of samples for species *i*, and *n*_*max*_ the number of samples available for the species with the most samples. The specimen images with removed background are padded to square format. Then, a random square region containing at least 70% of the image is selected and resized to 224x224 pixels, as our MobileNetV3 backbone network was also trained on images of this size. In addition, brightness and contrast of the image were jittered by ±10%, and saturation by ±5% to make the network more robust against slight changes in illumination and to remove lighting conditions as a potential confounding variable. Gaussian noise with a mean of 0 and a standard deviation of 0.05 was applied to the image to introduce additional variance in the training dataset. Finally, images were rotated by a random amount, to encourage the network to learn feature locations relative to their position on the shell rather than to their image coordinates and to avoid having slight variances in shell rotation as a potential confounding variable. We explicitly avoided the common technique of flipping images, as this would transform the images of dextral shells into pseudo-sinistral ones. Since such snails actually exist in nature, this image transformation could have serious biological implications, especially if a future dataset contained both dextral and sinistral shells. Shell measurements were augmented by multiplying them with Gaussian noise with a mean of 1 and a standard deviation of 0.025, adding variability and reducing overfitting. Data were normalized by prepending a batch norm layer to the network. For testing, images were resized to 224x224 pixels, but no further augmentations were applied to images, shell measurements, or collection site metadata.

Classification-only models and SSL embeddings were trained on randomly sampled batches. For supervised embeddings we follow the approach outlined in [61], where for a training batch first 16 classes are selected at random, and then the batch is constructed from four random samples from each of the selected classes. Using this class-balanced sampling for supervised embedding learning was shown to produce more meaningful embeddings [61]. We select the number of class-balanced batches so that supervised embedding models see the same amount of data as the other models during each training epoch.

### Evaluation

In our experiments, we measured performance using the macro-averaged F1 score, as well as top-3 accuracy on the test dataset. F1 score is the harmonic mean of precision and recall, defined as


$$\begin{array}{c} \begin{array}{c} F_{\text{1}}=\text{2}*\;\frac{precision\cdot recall}{precision+recall}=\frac{\text{2}\cdot TP}{\text{2}\cdot TP+FP+FN} \end{array} \end{array}$$ (4)

with number of true positives TP, false positives FP, and false negatives FN. The macro-averaged score is then the arithmetic mean of the per-class scores. Macro-averaged F1 score has a value between 0 and 1. A high score indicates that the system consistently predicts the correct species, even for specimens from underrepresented species. This provides a better performance measure than accuracy, as it gives equivalent weight to both precision and recall. In addition, with macro-averaging low performance on a species with few samples cannot be overshadowed by the high performance of a species with many samples.

In addition, we evaluated the top-3 accuracy of our models, which is the fraction of predictions, where the correct species is among the three predictions with the highest confidences. This metric is reflective of a real-world setting, where the system is not used as a replacement for a taxonomist, but instead as a support system that provides a short list of three likely candidates for the taxonomist, so they can restrict their efforts to likely species.

Following [63,65,75], we evaluated the model performance by training a 3-Nearest Neighbor (KNN) classifier on the training dataset embeddings. After training, we computed the embeddings for all training and test samples. Test samples are then assigned to the class that occurs most frequently among their three closest training samples in the embedding space. This allows us to directly evaluate the learned similarity metrics. As an analog to top-3 accuracy in this setting, we used the neighbor hit rate, which is the fraction of cases where at least one sample with the correct species is present among the three nearest neighbors.

In each of our experiments, we split the data into three disjoint sets: training, validation, and testing. After each training epoch, the model performance was evaluated against the validation data, and after training, the model with the best performance on the validation data was evaluated against the test set. This also helps mitigate potential overfitting, where the model still improves performance on the training set, but fails to generalize to different data. We used 5-fold cross-validation stratified by class for data splitting and repeated it five times with different splits to obtain more robust results.

## Results

### Performance on known sites

**Fig 5:**
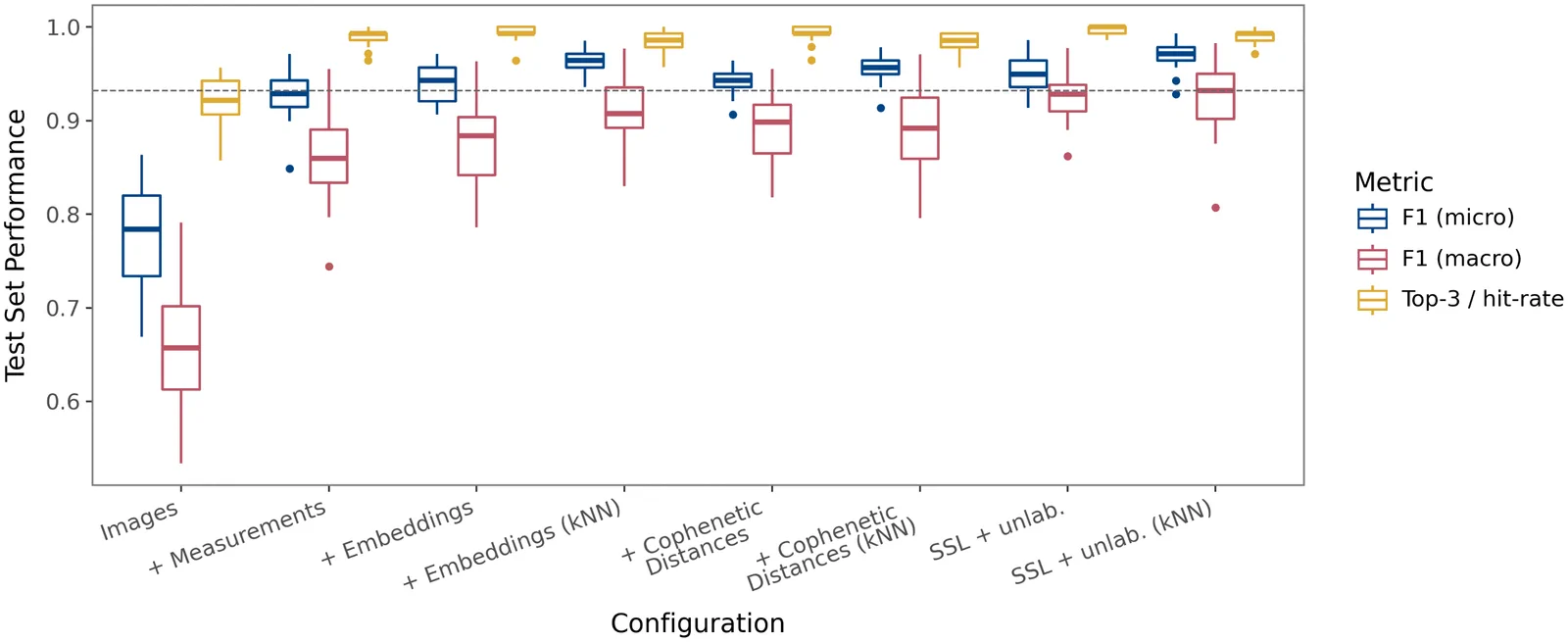
Test-set classification performance for different model configurations. The dashed line marks the highest median macro-averaged F1 score. Including shell measurements and collection site metadata alongside images improves system performance, learning embeddings and classification simultaneously further increases performance. Integrating genetic information through the dynamic margin did not yield significant changes; self-supervised learning and the inclusion of unlabeled test data produced the largest improvements.

As a first experiment, we evaluated how well the system performs using various combinations of images, measurements, collection site metadata, and genetic information. The baseline is the network trained on images alone, without using measurements or learning embeddings. We compared this baseline with a multimodal network trained on images and measurement data, and with an embedding network trained on images and measurement data with the combined loss function from Equation (3). Embedding networks were trained once with a static margin, and once with the dynamic margin that includes genetic information. Fig 5 shows the performance of the different configurations.

Learning from images and measurements led to far better performance than learning from images alone; and optimizing for embeddings and classification further improved performance over optimizing for classification alone. Integrating genetic information with the dynamic margin did not improve classification performance over embeddings learned without dynamic margin, but self-supervised learning of embeddings and the inclusion of unlabeled test data did.

**Fig 6:**
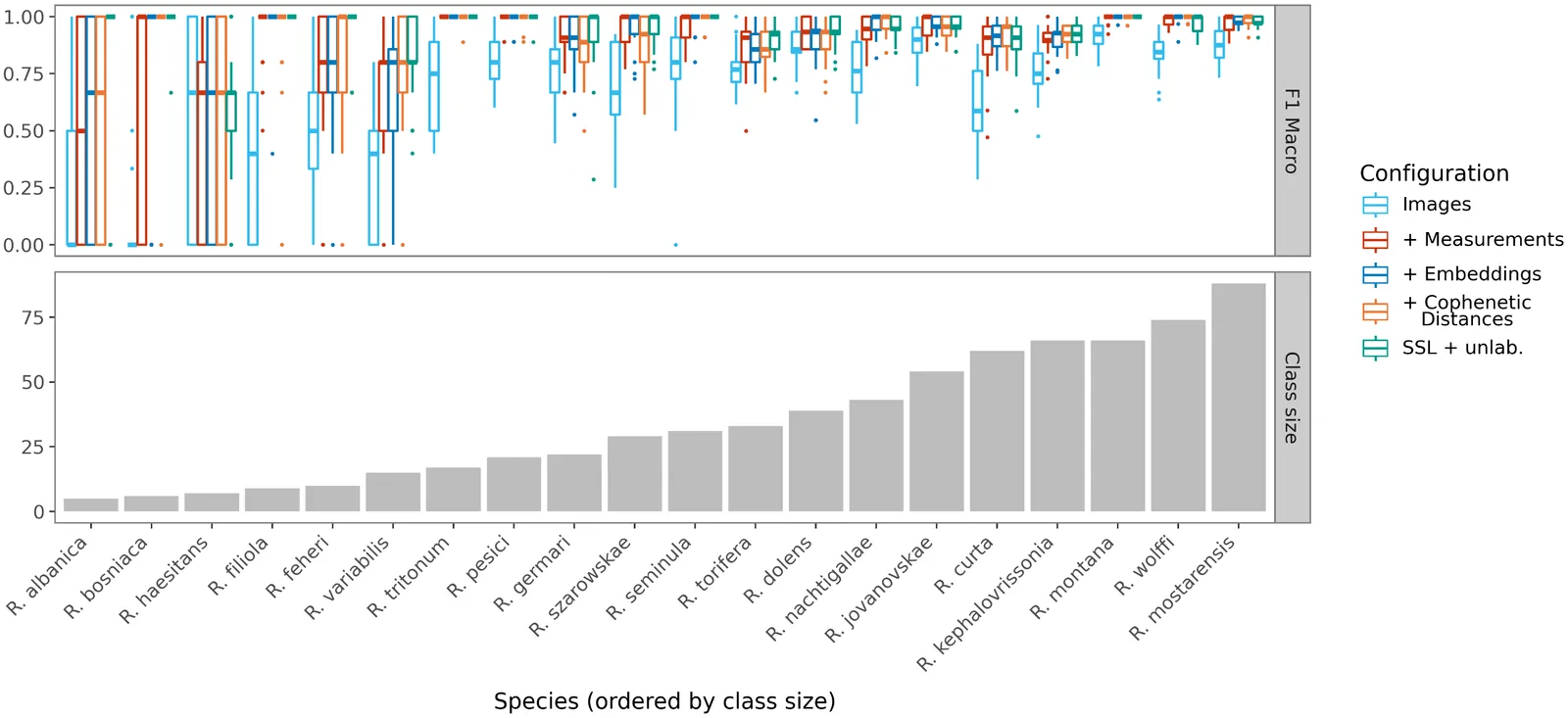
Class-wise test-set classification performance for different model configurations. Variance was very high for classes with few samples, spanning from 0 to 1. On the larger classes the models have a consistently good performance.

We also observed large variances in the macro-averaged F1 score across all methods. This variability is likely attributed to the limited number of samples available for certain species, resulting in test sets containing only one or two specimens. Consequently, the F1 score is either 0 (incorrect prediction) or 1 (correct prediction). With 20 species in total, missing a single prediction can therefore result in a drop of approx. 0.05. Indeed, when we investigated the distribution of F1 scores by class size (Fig 6), we observed that the smaller classes had a much higher variance, spanning from 0 to 1, whereas the performance had consistently high values for larger classes. Only models incorporating self-supervised embeddings and unlabeled test data consistently achieved reliable performance in classes with very few available samples.

### Different methods for embedding learning

In recent years, multiple improvements over Siamese networks have been proposed [61]. We therefore want to evaluate the influence of the specific method for embedding learning on the overall performance of our system. Each new method proposes a different way of sampling candidates (Siamese networks typically use random tuples), a different loss function to the one described in Equation (1), or both. We compared 8 different methods that achieved the best results in a previous comparison study [61], namely: (1) random tuples with the contrastive loss, (2) triplets with hard positives and semi-hard negatives with the triplet margin loss [52], (3) triplets with easy positives, semi-hard negatives and the loss function proposed in [57], (4) triplets with learned margins [60], (5) the SoftTriple loss proposed by [58], (6) embeddings learned with normalized softmax [76], (7) CosFace [77], and (8) ArcFace [59]. For the contrastive loss and triplet margin loss we also include variants that use a dynamic margin incorporating genetic information. Method (1) is the Siamese network approach described earlier. Methods (2)-(4) are variations of triplet networks. In this context a “positive” refers to a different specimen from the same species, while a “negative” refers to a specimen from a different species; “easy” or “hard” refers to how similar the specimens seem. For example, a hard positive is a same-species pair, that nonetheless looks quite different, and a semi-hard negative is a different-species pair that looks fairly similar. Overall, Methods (1) – (4) compare samples to each other, while Methods (5) – (8) compare samples to learned representatives. In addition, methods (4) – (8) have internal learnable parameters, for example a class-specific margin penalty in [60]. These internal parameters were trained jointly with the rest of the network, using the same optimizer and training settings. All methods also have non-learnable hyperparameters, such as the target margins between classes. We did not perform a search for optimal values for these hyperparameters, but instead used the values suggested by [61,78].

In addition, we also compare multiple self-supervised methods: (9) self-supervised contrastive learning with the InfoNCE loss introduced in Equation (2) [62,64], (10) BYOL [63], and (11) DINO [65]. All three of these methods work by aligning the embeddings belonging to different views of the same input. Each of these methods is evaluated with and without inclusion of unlabeled training data to investigate if integrating additional unlabeled data improves performance.

**Fig 7:**
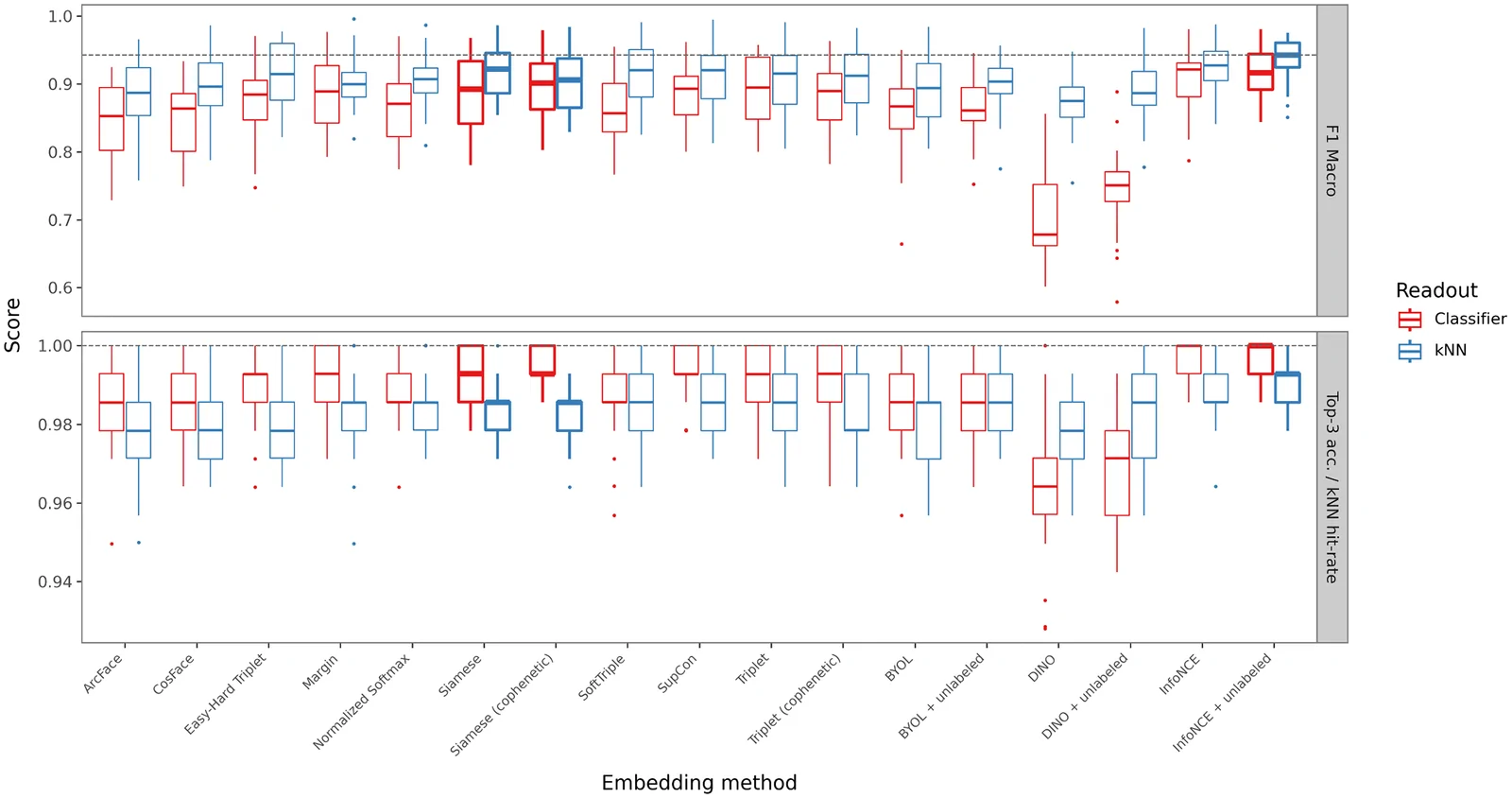
Test set classification accuracy with different ways of learning embeddings. The dashed line marks the highest median macro-averaged F1 score. Embeddings learned with the contrastive loss function or the InfoNCE loss and unlabeled test data are among the best performing.

Overall, the supervised methods have a comparable performance, with the contrastive loss (Siamese) among the best performing. The self-supervised methods differ significantly, with the self-supervised InfoNCE embeddings clearly outperforming the other two methods and all self-supervised methods benefiting from the inclusion of the unlabeled test data (Fig 7). Because we did not specifically tune hyperparameters, some methods may be slightly underperforming. Rather than invest in an extensive search, we proceeded with the supervised contrastive loss and the self-supervised InfoNCE, due to their simplicity and effectiveness.

**Fig 8:**
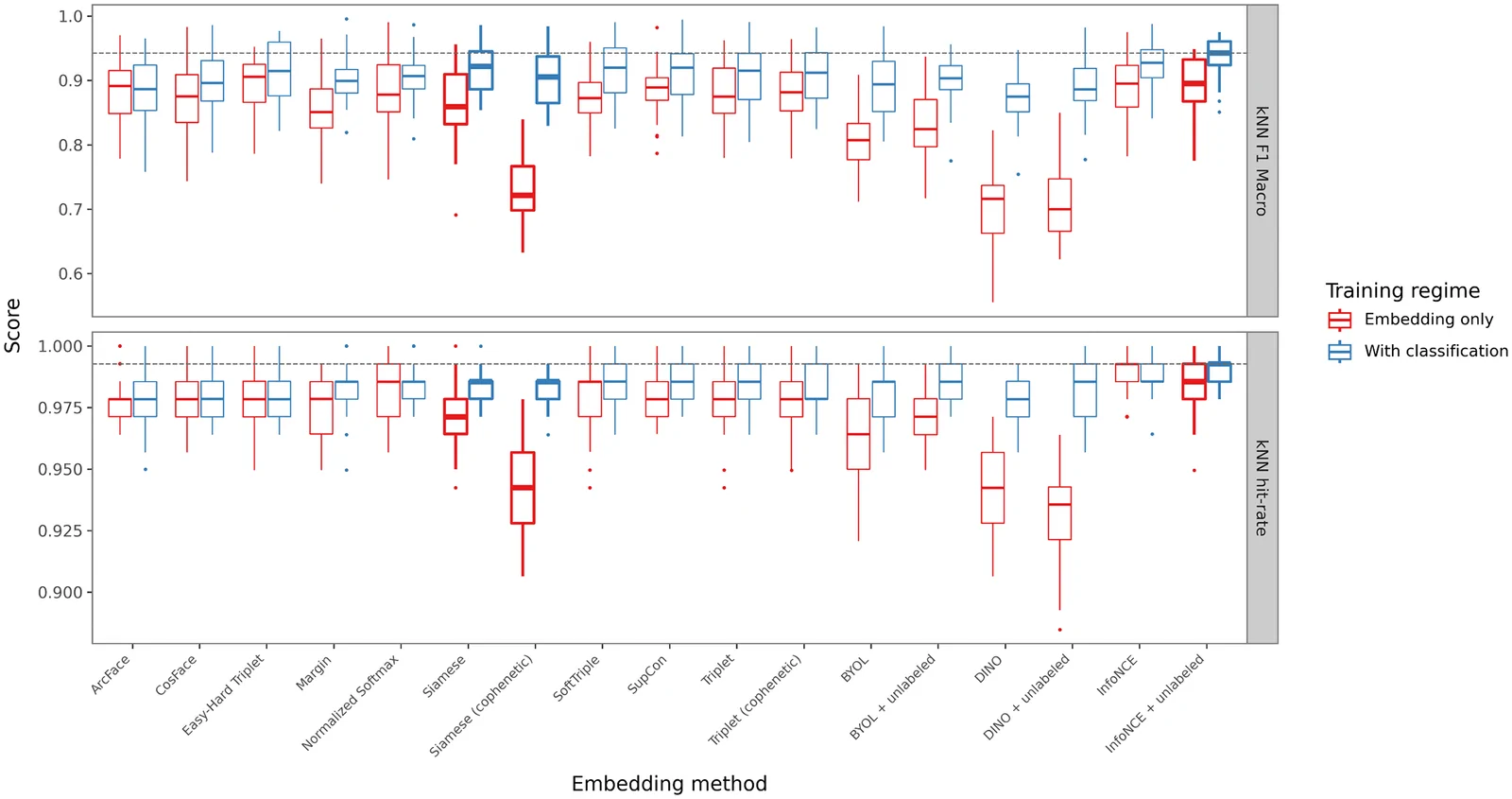
Test set KNN classification accuracy on embeddings trained with and without an additional classifier. Highest median performance is marked with a dotted line. Joint training of embeddings and classification consistently leads to better performing embeddings than training embeddings alone.

Interestingly, we observe that top-1 performance across all methods is higher if the trained classifier is ignored and instead a KNN classifier is applied to the embeddings. In contrast, for top-3 performance, it is the opposite, with top-3 accuracy higher than the KNN neighbor hit rate. A possible explanation for this phenomenon could be that top-3 accuracy always evaluates against three different candidate species, while the three closest neighbors might belong to only one or two different species. Due to the superior performance of the KNN classifier, we next investigate if joint learning of embeddings and classification provides an advantage over learning embeddings alone (Fig 8). To make the results comparable we evaluate embeddings in both cases with a KNN classifier. We observe that including the additional classifier in the training improves quality of the embeddings for the downstream classification task, even if its output is discarded.

### Performance on unseen sites

**Fig 9:**
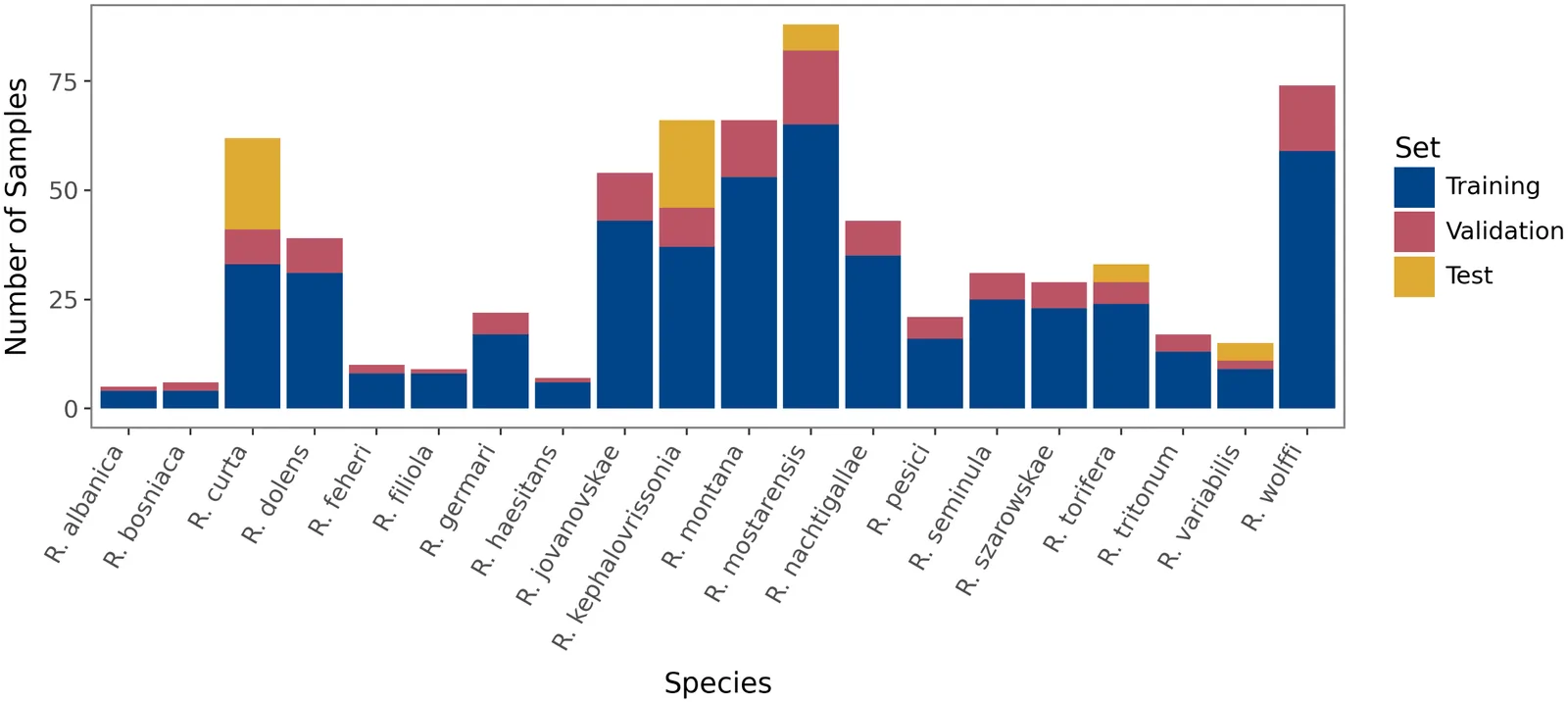
Species distributions for training, validation, and test set. Specimens from five species and six collection sites are selected for testing. The remaining specimens are split into five folds, stratified by species; four folds are used for training, one for validation.

In an additional experiment, we aimed to investigate how the system performs when classifying data from previously unseen collection sites – a realistic scenario where the system is used alongside a taxonomist to classify new specimens. For this, we split the dataset by collection site, ensuring all samples from a given site were included either entirely in the training dataset or entirely in the test dataset. For the test dataset, we selected species found at multiple sites; for each, we selected the sites with more specimens for training and the remaining ones for testing. Overall, the test dataset for this experiment consisted of 55 specimens from 5 different species and 6 different collection sites, with the species distribution shown in Fig 9.

Similar to our previous experiment, we split the training data into training and validation with a five times repeated five-fold cross-validation split. The model from the epoch where performance was highest on the validation set was then evaluated on the test dataset. We compared the performance of the network trained on (1) images, (2) images and measurements, and (3) images, measurements, and supervised embeddings (with and without genetic information), (4) images, measurements, self-supervised embeddings and unlabeled test data. Based on our previous findings, we evaluated supervised embeddings using the contrastive loss with and without dynamic margin, as well as the self-supervised InfoNCE embeddings. We omitted other methods for embedding learning as they did not provide significant further improvements.

**Fig 10:**
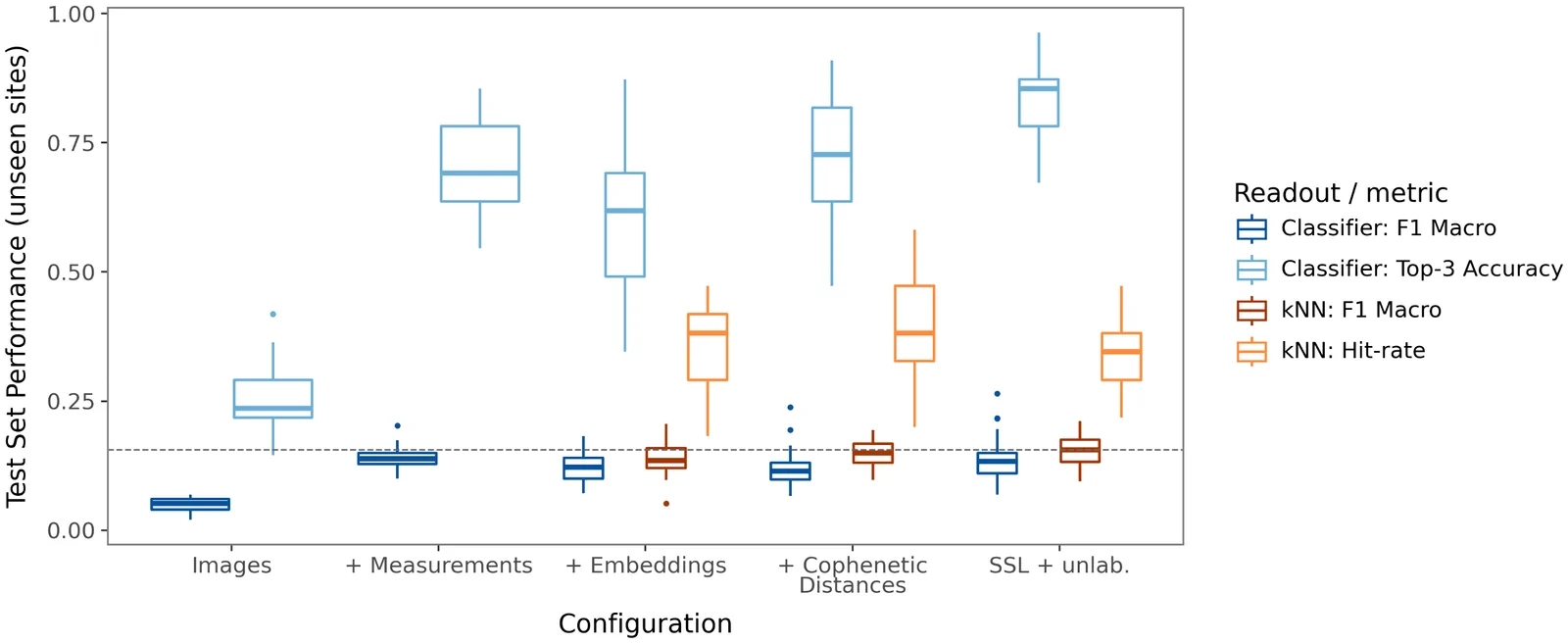
Test set performance for different configurations. The dashed line marks the highest median macro-averaged F1 score. Performance is significantly lower than on data from known collection sites. Including measurements improves performance, as does learning embeddings and classification simultaneously.

The results show that the previously observed trend holds: adding measurements improves performance, as does training embeddings and classification simultaneously (Fig 10). Again, adding dynamic margins does not significantly impact performance. However, there are two interesting observations.

The first observation is that across all configurations, performance drops drastically when tested on specimens from collection sites not present in the training set. Again, the best performance is achieved by joint optimization of classification and contrastive embeddings and the inclusion of unlabeled test data. However, the advantage over classification on multimodal data is much lower. Baseline mean F1 macro score for the multimodal classifier drops from 0.86 ± 0.05 to 0.14 ± 0.07 and the mean top-3 accuracy dropped from 0.99 ± 0.01 to 0.71 ± 0.09. Including self-supervised embeddings and unlabeled test data can improve this to a mean F1 macro score of 0.16 ± 0.03 and a top-3 accuracy of 0.82 ± 0.07, but still far below the performance on data from collection sites seen during training. In addition, learning supervised Siamese embeddings did not improve performance over the baseline and after Holm correction [79], neither method provided significant improvements over the baseline.

**Fig 11:**
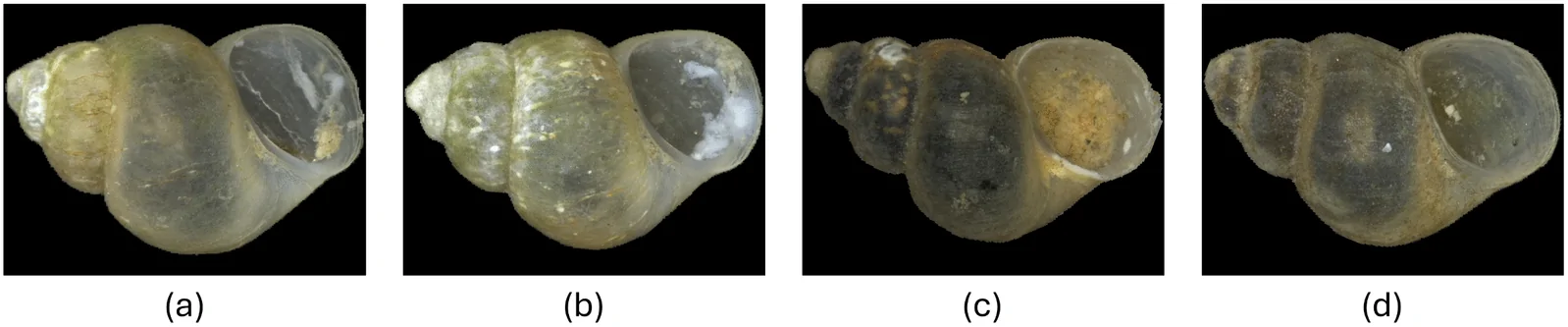
Intraspecific and interspecific variation across collection sites. a, b: *R. curta*, collected at Site A, c: *R. curta* collected at Site B, d: *R. jovanovskae* collected at Site D**.** Samples of the same species collected at the same site (a, b) are much more similar than samples of the same species collected from different sites (a,c). To an untrained observer, variance within the same species (a,b,c) can be almost as large as variance between species (a,b,d).

We then investigated possible causes of the overall much lower performance on data from collection sites not seen during training. A qualitative visual inspection found that specimens from the same collection site display high visual similarity, but, as is typical for cryptic species, intra-species variance of specimens from different collection sites can be significant. In some cases, this intra-species variance is almost as large as inter-species variance, particularly to untrained observers (Fig 11).

### Performance on unseen species

As a follow-up experiment, we performed a qualitative analysis of how availability of data from the same species influences system performance. For this we manually selected three collection sites as a holdout test set. The first site contained specimens from *R. curta,* for which the training dataset then included data from five other collection sites. The second site contained specimens of *R. kephalovrissonia,* which only occurred at one other collection site. Finally, the third site contained *R. tritonum* specimens, which were only found at this collection site. We trained three models to compare: (1) a multimodal classifier, (2) a multimodal classifier with Siamese embeddings, and (3) a multimodal classifier with self-supervised InfoNCE embeddings also trained on the unlabeled test data. Again, we split the training set into 5 splits, using 4 for training and one for validation. Models were trained for 20 epochs, then the model from the epoch with the best performance on the validation set was evaluated on the test set.

**Fig 12:**
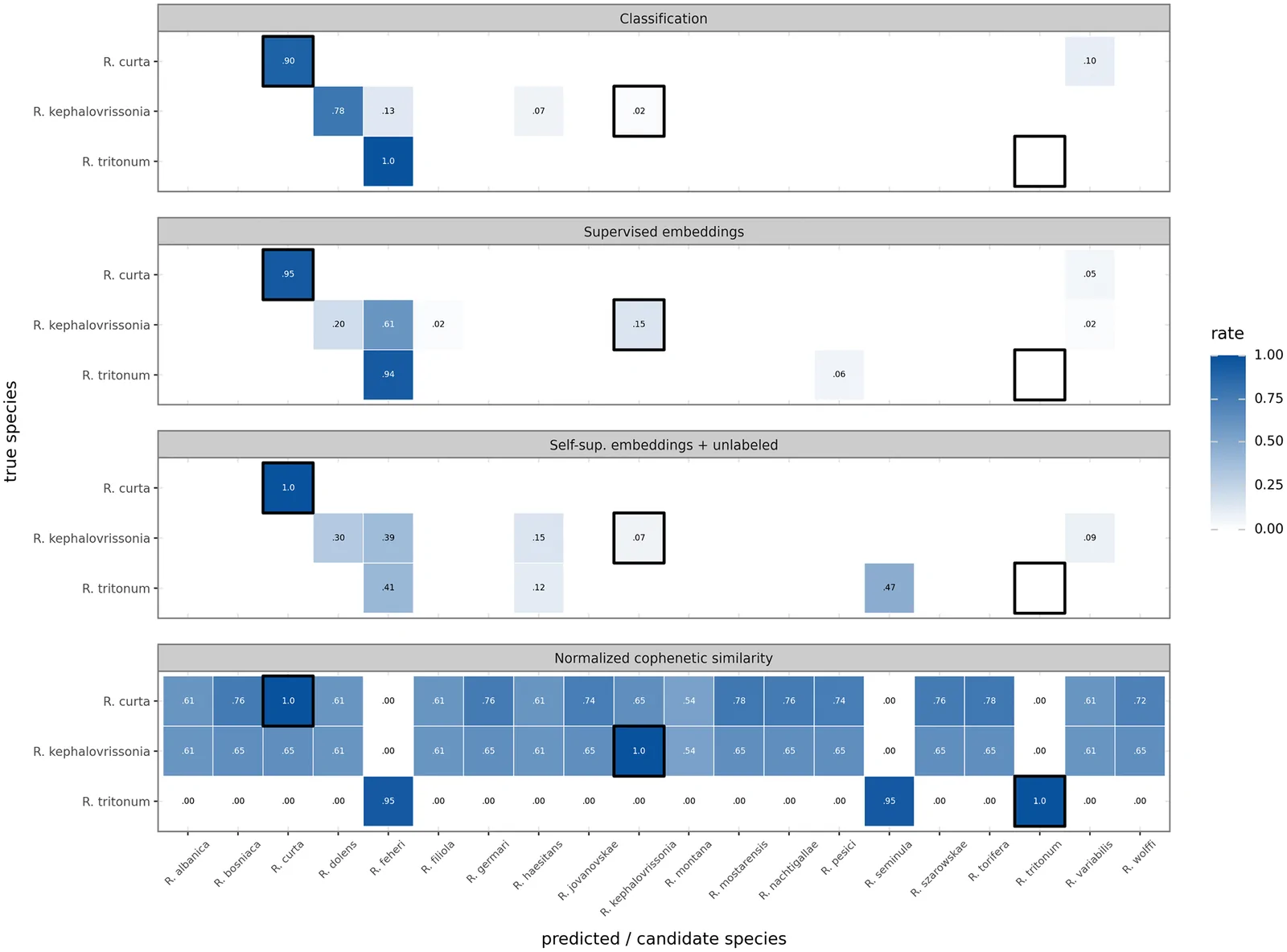
Confusion matrix of model predictions for known and unknown species. All models reliably identify *R. curta,* but have problems identifying *R. kephalovrissonia.* The pattern of model confusion remains largely uniform across different models, with incorrect predictions being assigned to the same set of candidate species. The unseen class corresponding to *R. tritonum* is mapped to cophenetically close known species.

First, we investigated how well the different models were able to predict the correct species (Fig 12). All models reliably identified *R. curta* but mostly failed to identify *R. kephalovrissonia.* Interestingly, the three models exhibited similar errors, with a highly analogous set of candidate species for incorrect predictions. Another interesting observation is that most specimens from the unseen class corresponding to *R. tritonum* are mapped to phylogenetically close species, whereas this is less frequently the case for *R. kephalovrissonia*.

**Fig 13:**
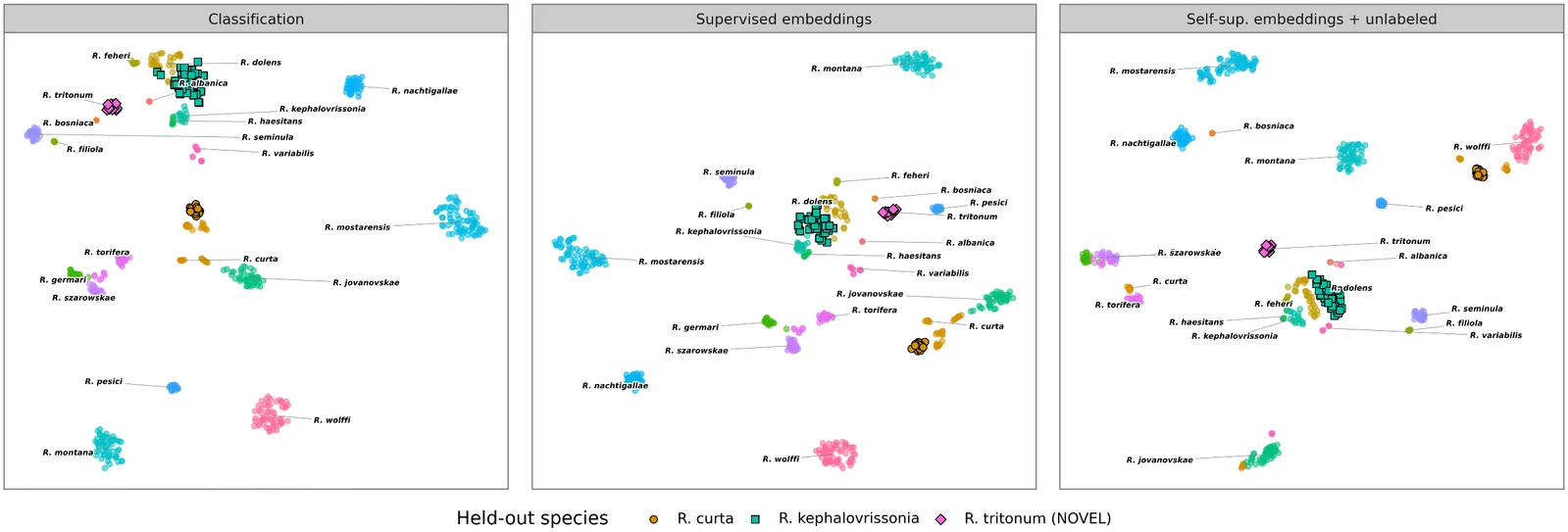
Two-dimensional t-SNE visualization of the 128-dimensional embeddings. Specimens from different species form mostly well-separated clusters, in both training and test data. Number of clusters corresponds to number of collection sites, indicating high intra-species variability. Even specimens from novel species form a clearly separated cluster.

Next, we used t-SNE [80] to visualize a two-dimensional projection of the 128-dimensional embedding space (Fig 13). Interestingly, the embedding spaces of the three models exhibited similar structure. Importantly, even in absence of explicit optimization, the multimodal classifier already produces meaningful embeddings. In all models we see a number of well-separated single-species clusters. However, the number of clusters aligns with the number of collection sites rather than the number of species. This is most visible in *R. curta*, which has the highest number of collection sites. Each collection site results in a clearly separated cluster of embeddings, but the different clusters are spread out, highlighting substantial intra-species variability. Only the supervised Siamese training pulls these clusters closer together. Similarly, the specimens from *R. variabilis* exhibit considerable intra-species variability, resulting in widespread distribution across the embedding space when labels are not included (self-supervised training). Another observation is that the high visual similarity between some *R. curta* and *R. jovanovskae* specimens illustrated in Fig 11 is mirrored in the embedding space by the closeness of an *R. curta* cluster to the *R. jovanovskae* cluster. Finally, the specimens for the unseen class corresponding to *R. tritonum* consistently formed a distinct cluster separate from other species in all three models.

**Fig 14:**
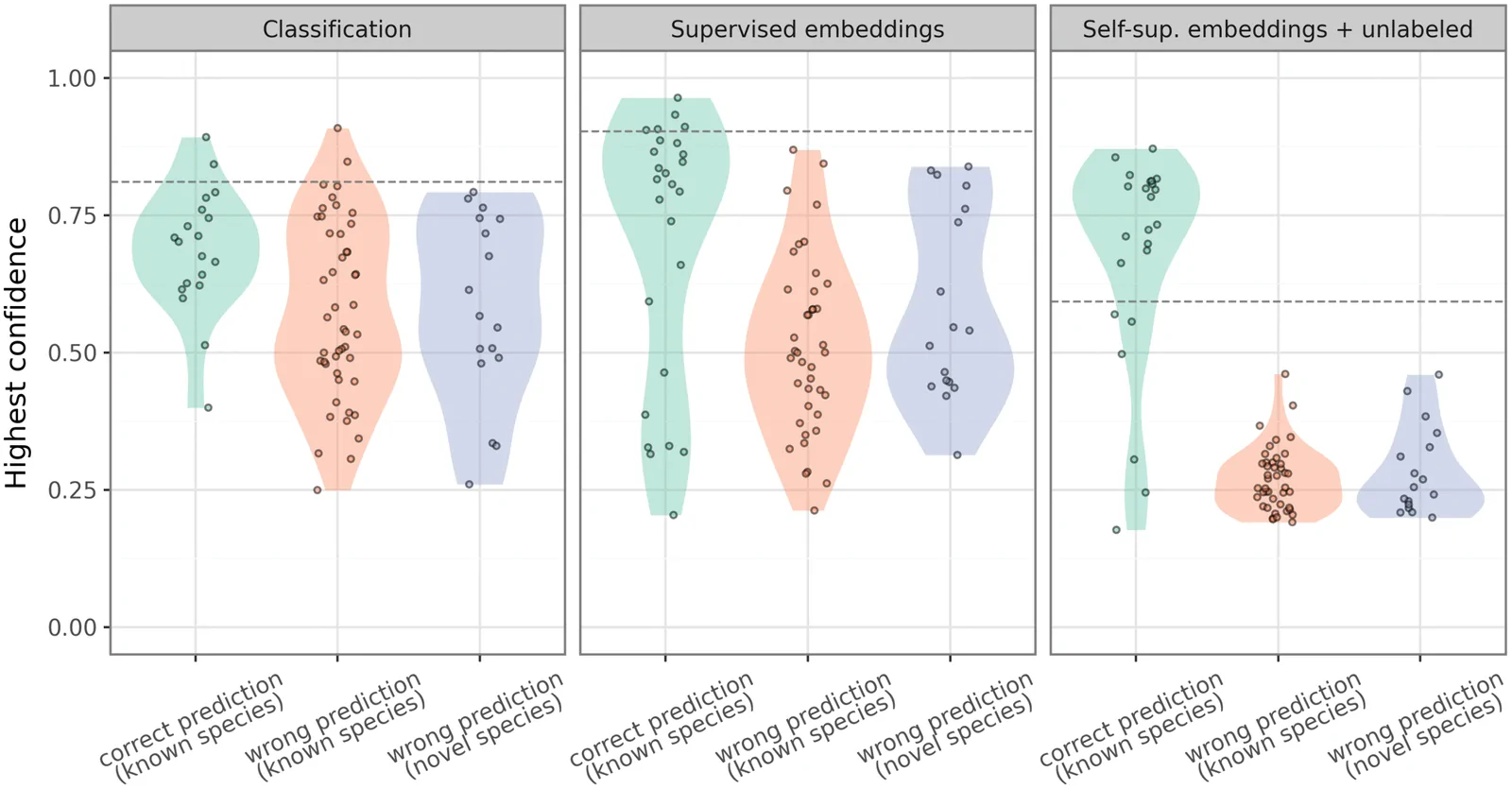
Comparison of maximum confidence outputs for three different models. The dotted line marks the confidence threshold computed using Youden’s J statistic on the validation dataset. The model trained on classification alone often confidently predicts an incorrect class. The model trained with classification and supervised embeddings tends to have high confidence in correct predictions and low confidence in incorrect predictions. The model trained on classification, self-supervised embeddings, and the inclusion of unlabeled test data was the best calibrated. High-confidence predictions are always correct, low-confidence predictions are almost always incorrect, and the confidence threshold identified with Youden’s J statistic on the validation set provides a useful heuristic to identify incorrect predictions.

We also analyzed the models’ prediction confidence on the test data. We used temperature scaling [81] for confidence calibration on the validation set to reduce the error between model output confidence and probability of correctness. In addition, we used Youden’s J statistic [82] on the validation set to identify the confidence threshold where the difference between true positive rate and false positive rate is maximal. We then compare the predictions (output class with highest confidence) of the three models (Fig 14).

We see that the model trained on classification alone performs worst. It frequently makes confident predictions, even when the predictions are wrong. In addition, the confidence threshold from the validation set does not transfer well to the test set. The model trained with supervised embeddings and classification performs better, correct predictions tend to have a higher confidence than incorrect predictions, and while not many test samples produce confidence values above the validation confidence threshold, it translates better than in the classification case. The model trained on self-supervised embeddings, classification, and the inclusion of unlabeled test data performs best. Incorrect predictions have low confidence, for both known and unknown classes, while most of the correct predictions have a much higher confidence. In addition, the confidence threshold identified on the validation set transfers very well to the test dataset, providing a useful heuristic for the identification of incorrect predictions.

**Fig 15:**
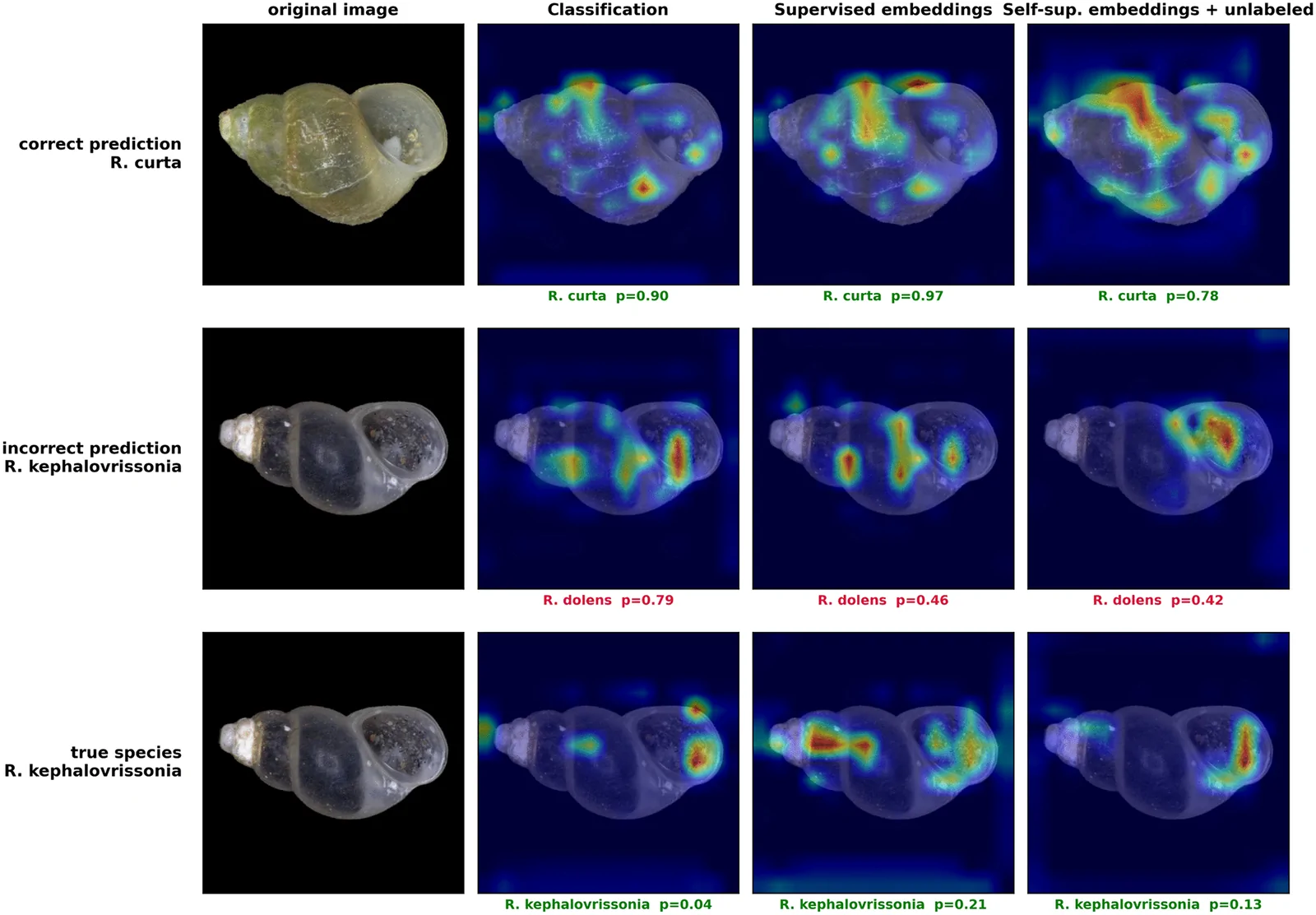
GradCAM visualization of image regions relevant to the prediction. We compare three cases (1) the model makes the correct prediction, (2) the model makes an incorrect prediction, (3) the relevant regions for the actual class if the model makes an incorrect prediction. All models focus on relevant parts of the shell. Inspecting activations does not provide a clear heuristic for recognizing incorrect predictions.

Lastly, we use GradCAM [83] to investigate which image regions the model uses for its prediction. We qualitatively compare three cases: (1) the most confident species when the model makes the correct prediction, (2) the most confident species when the model makes the incorrect prediction, (3) the actual species when the model makes an incorrect prediction (Fig 15).

We see that all three models look at similar shell regions to inform their prediction. In addition, almost all of the relevant regions are on the whorls, aperture, or the apex of the shell, with minimal emphasis placed on the background.

### Performance with incrementally available labels

Based on the good performance demonstrated on data from collection sites seen during training and the bad performance on data from collection sites not seen during training, we finally investigated how much labeled data from a collection site the system needs for good performance. This simulated the use case in which taxonomists collect specimens from a new site and then use the system to support their work. Initially, no correct labels for the samples were available, but as the taxonomists perform their work, new labels gradually become available. We aimed to assess how having incrementally more labels available influences the system’s performance on data from new collection sites.

One site we selected for evaluation has samples from *R. curta,* as this is the species encountered at the most distinct sites in our dataset. Our selected site consists of 20 individuals, while the other four sites have 10 or 11 individuals each. We also selected a site with individuals of *R. kephalovrissonia*, which was only encountered at two distinct collection sites, but has a larger sample size at those sites. The selected site consists of 46 samples, and the other site consists of 20. With these two sites, we can investigate whether and how the availability of data from multiple sites influences performance on specimens from new sites.

To evaluate performance on a site, we use a five-fold cross-validation with four folds used for training, and one for validation. We then iteratively moved labeled samples from the test set to the training set, trained the system for 20 epochs, and evaluated performance on the remaining test samples with the model snapshot from the epoch that performed highest on the validation set. This procedure was repeated five times with different splits to obtain more robust results. Training our system for 20 epochs on the whole dataset required less than five minutes.

**Fig 16:**
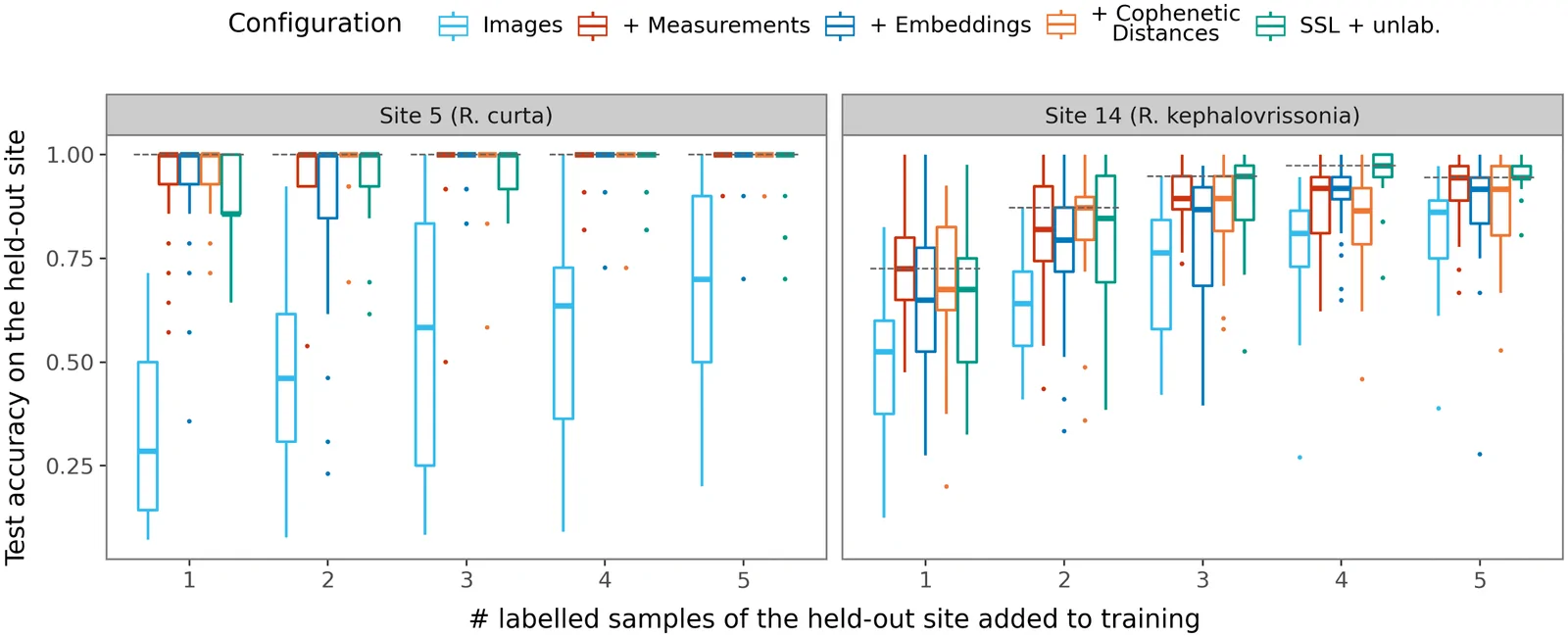
Performance on unseen sites with incrementally added labeled samples. The dashed line marks the highest median accuracy score at each retraining. Inclusion of shell measurements and collection site metadata significantly improves performance. In contrast, the different methods for additional embedding learning only have minimal impact.

As in previous experiments, the inclusion of shell measurements and collection site metadata provides the single largest improvement to model performance, while joint optimization of embeddings and classification only provided minimal additional impact (Fig 16). We see that for both species only five additional samples from a new collection site allow the models to reliably learn the relevant patterns for identification. In addition, for *R. curta* good performance is already achieved with only one additional labeled sample, while for *R. kephalovrissonia* additional data is required.

### Reduced dataset size

**Fig 17:**
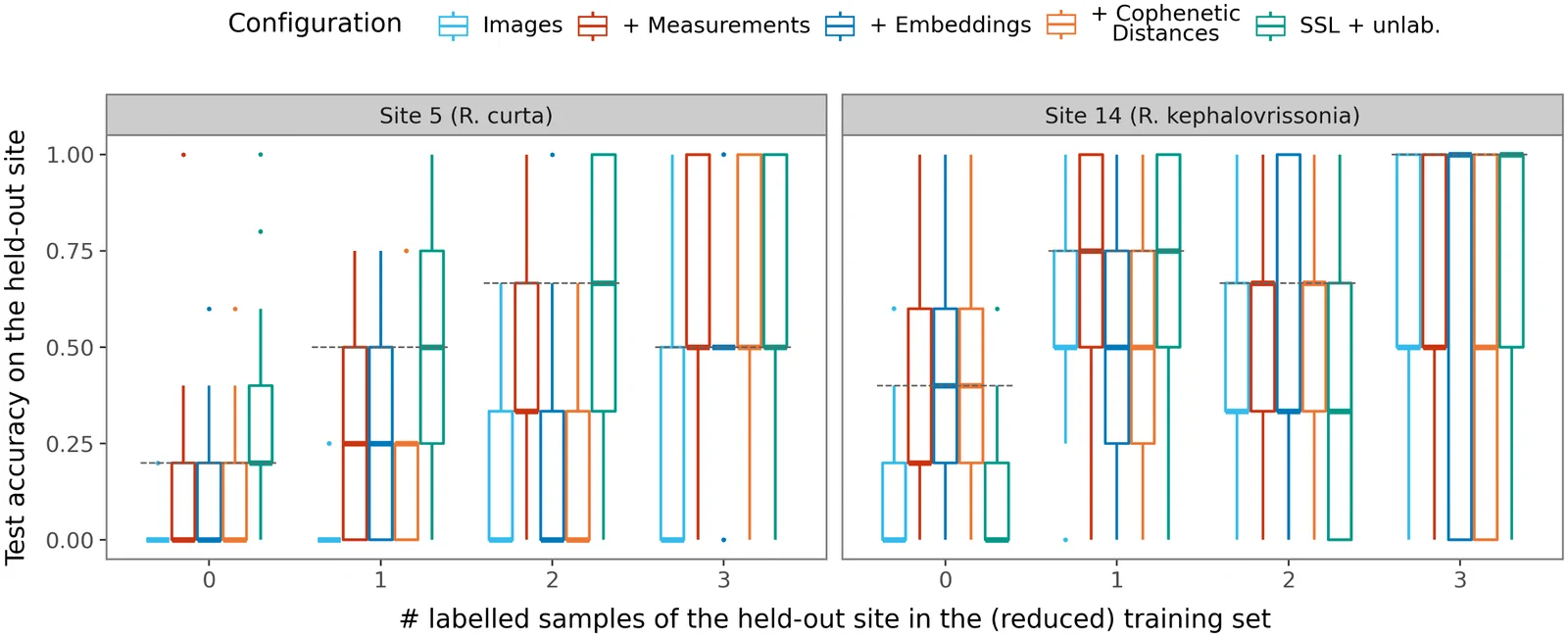
Performance on unseen sites with small training datasets and incrementally added labeled samples. The dashed line marks the highest median accuracy score at each retraining. Variance in outcomes is very high for all models. The model including self-supervised embeddings and unlabeled test data seems to perform slightly better than the others.

Our dataset contained approximately 700 samples. As most datasets available in taxonomic use cases are of smaller size, we were interested in learning how our approach performs with a much smaller dataset. To reduce dataset size, we selected up to 2 collection sites per species, with up to 5 specimens per site. This random sampling reduced the training dataset size to 128 when testing with *R. curta* and 118 when testing with *R. kephalovrissonia*, which is much more in line with common sizes of taxonomic datasets. In addition, for testing, we also selected 5 random samples from the test site. Similar to our previous experiment we conducted a five fold split on the training dataset. We trained on four splits and used one for validation. We also iteratively moved labeled samples from the test dataset to the training dataset, retrained our system for 20 epochs, and used the model from the epoch with the best performance on the validation dataset to evaluate performance on the test dataset. This procedure was then repeated five times with different splits to get more robust results. In contrast to earlier findings, the results display much larger variance (Fig 17), and there is no clear best-performing model. However, the model including self-supervised embeddings and unlabeled test data (comprising the remaining 2–5 samples from the downsampled test dataset) appears to perform marginally better than the others. In addition to achieving commendable accuracy, it attains a median top-3 accuracy of 1.0 with the inclusion of only two additional samples.

## Discussion

Species in the cryptic freshwater snail genus *Radomaniola* can be identified with high accuracy from a combination of shell images, morphometric measurements, and basic collection-site metadata, despite their very subtle morphological differences that challenge even specialists. This accuracy was achieved under conditions that are typical, and often prohibitive for taxonomic datasets; namely a small number of samples and a strong class imbalance. We obtained these results with a relatively simple joint optimization of self-supervised and supervised training, demonstrating that data-efficient similarity learning is a viable route to computer assistance of demanding taxonomic workflows.

### Multimodal inputs and similarity learning improve identification of cryptic taxa

Two aspects of the approach were particularly important. Combining shell images with morphometric measurements and basic collection site metadata consistently improved performance over image‑only models. Most computer-vision approaches to cryptic species identification have relied on images alone, whether of wall lizards [14], coral reef fish [15], limpets [16,17], whiteflies [18], mosquitoes [19], or nematodes [20]. Our results show that even the inclusion of basic morphometric measurements and locality data provides a consistent improvement upon this standard, indicating that the information taxonomists routinely record alongside their specimens is worth encoding directly in the model. Jointly learning specimen embeddings and species labels further increased accuracy and stability (Fig 5). This is consistent with earlier work showing that similarity learning is well suited to the small, imbalanced datasets typical of taxonomy, for example for the identification of plants [22], snakes [23] or fish [21]. Our study therefore provides additional evidence that similarity learning provides a valuable tool for mitigating the limitations of taxonomic datasets. Among the tested metric‑learning strategies, simple self-supervised embeddings using the popular InfoNCE objective [62,63,65,84,85] provided the most robust performance (Fig 7). In our system the combination of this self-supervised embedding learning and the classification objective allows us to exploit both available labeled samples and additional unlabeled data for optimal performance (Fig 8). This is especially relevant to taxonomic workflows, as it further lessens the requirement for large, labeled datasets for the efficient application of deep learning methods. In addition, models trained with self-supervised embeddings are well calibrated, allowing the identification of incorrect predictions through their low prediction confidence (Fig 14). Finally, in contrast to recent large-scale multimodal efforts that combine images with DNA data across millions of specimens [86–89], our results show that self-supervised similarity learning strategies remain effective in low-data regimes, without genetic input or extensive tuning.

### Morphological variation in *Radomaniola* is structured by population, not species

Performance dropped significantly when the system was applied to specimens from collection sites not seen during training, even though top‑3 accuracy remained high enough to offer useful candidate lists (Fig 10). A likely cause of this drop in performance is that the model’s learned similarity metric clusters specimens at the collection-site level rather than at the species level (Fig 13). This is likely not a model failure, as the model is able to reliably form distinct clusters for specimens from known species and new collection sites, as well as previously unseen species (Fig 13). This suggests that the model successfully learned to capture similarity between specimens. Therefore this behavior appears to reflect genuine biological structure as much of the phenotypic variation is organized at the population or site level. Conspecific populations from different springs can resemble closely related species just as much as they resemble each other (Fig 11). This mirrors recent findings in other shelled mollusks, where cryptic morphological divergence also aligned with genetically distinct populations rather than species boundaries [16]. It also suggests that the observed population level structuring of shell morphology is not unique to *Radomaniola* but may be a recurring challenge for the image-based identification of other taxa with strong geographic structure. Our study also offers a concrete instance of the geographic generalization gap that is increasingly reported in computer vision approaches to biodiversity, where models that perform well on seen site data degrade on data from new locations [90–92]. However, our results indicate that in cryptic taxa this gap corresponds to a genuine biological signal, rather than dataset biases, spurious correlations, or overfitting. Additional evidence against model failure is the model’s ability to cluster both specimens from known species and new collection sites, as well as unknown species (Fig 13). This emerging cluster structure at the locality level instead of species level, and the fact that adding phylogenetic information via a dynamic margin did not significantly improve results, suggest that, for *Radomaniola*, molecular data are not essential to building a practical classification system once good images, shell measurements, and locality data are available. This also reinforces previous observations of morphostatic evolution in *Radomaniola* [25], where morphological change is decoupled from phylogeny.

### Annotation efficient adaptation enables practical deployment

From an applied perspective, the most encouraging result is the rapid recovery of performance when a few labeled specimens from new sites are added (Fig 16). Retraining with one or two newly identified specimens already led to substantial gains, and additional labels improved accuracy smoothly and predictably, even when the overall training set was reduced to sizes typical of many taxonomic studies (Fig 17). Incremental learning has previously been used in wildlife image classification to mitigate the location gap, accommodate previously unseen species, and incorporate newly annotated samples as they become available [93–95]. The success of incremental learning also connects back to the previously discussed site-level population structure. While this population structure degrades transfer to unseen collection sites, it is efficiently mitigated once a few labeled specimens anchor the new population. With just a handful of newly annotated specimens, the model can rapidly adapt to new localities and continue to support taxonomic work.

### Limitations

While our results demonstrate strong performance across multiple realistic scenarios, several limitations should be considered. First, we evaluated our approach only with a single genus. Some findings may not transfer to other taxa, such as the organization of variation at the population level rather than the species level, or the observed limited importance of genetic data. Our *Radomaniola* dataset is also, taxonomically speaking, on the larger side. On the much smaller subsampled dataset that better reflects the size of typical taxonomic datasets, effects are less pronounced and show substantially higher variance. Furthermore, our approach relies on the availability of high-quality images, which limits its applicability under field conditions. In addition, our approach of one-hot encoding categorical variables, such as country of origin, cannot handle metadata unseen during training. Including specimens from a country absent from the training set would require retraining with re-encoded features. Another limitation concerns potential deployments. As indicated by the performance drop on unseen localities (Fig 10), our approach is not suited for static models that are trained once and then distributed to users; at least not in taxa like *Radomaniola,* where variation is largely organized at the population level. Although performance can be recovered through incremental learning with a small number of labeled samples from the new locality, this also implies that the system needs to be continually retrained as new information becomes available to remain useful to taxonomists. Fortunately, even on standard hardware full retraining (less than five minutes on an Nvidia GeForce GTX 1660 Super GPU with 6GB VRAM) remains far below the time required for a taxonomist to classify a new specimen. Finally, the detailed analysis of performance on unseen species was based on a single set of trained models. While the qualitative trends were reproduced across repeated training runs, the specific values should be read as indicative of these trends rather than as precise point estimates, and comparisons of small differences between conditions should be treated with corresponding caution.

## Conclusions and implications for taxonomy and biodiversity monitoring

In summary, our findings indicate that multimodal Siamese network models can be integrated into taxonomic workflows as effective decision‑support tools. They can be trained and retrained quickly on standard hardware, cope reasonably well with limited and uneven data, and can be updated as new determinations become available. While they do not replace anatomical or genetic analyses, they can reduce repetitive comparison work and help focus expert attention on the most ambiguous specimens and populations. Our approach is therefore best understood within an integrative-taxonomy framework [96–98], complementing other types of evidence rather than substituting for them. The model triages specimens and surfaces candidates, while the final determinations remain with the taxonomist.

More broadly, our results suggest that data-efficient, multimodal machine learning approaches can support biodiversity research even when available datasets are limited. This is particularly relevant given the well-recognized shortage of taxonomic expertise [6,9,10]. By enabling faster and more consistent specimen identification, and by remaining trainable on modest hardware and datasets, such systems may facilitate biodiversity monitoring and conservation efforts, particularly in settings where access to taxonomic expertise and resources is limited.

A further advantage of building the model around self-supervised similarity learning is that the embedding component does not, in principle, require labels and can be trained on unlabeled or only partially labeled specimens [62,99]. This opens the possibility of including large numbers of unlabeled specimens from collections or monitoring programs to shape the embedding space, while a comparatively small set of expert determinations anchors the species labels. Such an approach could further reduce the annotation burden and widen the applicability of the method to taxa and collections where labeled material is especially scarce.

### Declaration on usage of Large Language Models (LLMs)

During the preparation of this work the authors used ChatGPT 4.0/5.0/5.1/5.2 and Claude Opus 4.8 in order to help with the coding process and to improve language and readability. After using this tool, the authors reviewed and edited the content as needed to ensure the content is accurate and valid. The authors ensure all statements in the article reporting hypotheses, interpretations, results, conclusions, limitations, and implications of the study represent the authors’ own ideas and take full responsibility for the content of the article.

## References

[1] Neubauer TA, Hauffe T, Silvestro D, Schauer J, Kadolsky D, Wesselingh FP, et al. Current extinction rate in European freshwater gastropods greatly exceeds that of the late Cretaceous mass extinction. Commun Earth Environ. 2021;2(1). doi: 10.1038/s43247-021-00167-x

[2] Mamantov MA, Gibson‐Reinemer DK, Linck EB, Sheldon KS. Climate‐driven range shifts of montane species vary with elevation. Global Ecol Biogeogr. 2021;30(4):784–94. doi: 10.1111/geb.13246

[3] Diamond SE. Contemporary climate‐driven range shifts: Putting evolution back on the table. Functional Ecology. 2018;32(7):1652–65. doi: 10.1111/1365-2435.13095

[4] Schmeller DS, Böhm M, Arvanitidis C, Barber-Meyer S, Brummitt N, Chandler M, et al. Building capacity in biodiversity monitoring at the global scale. Biodivers Conserv. 2017;26:2765–90. doi: 10.1007/s10531-017-1388-7

[5] Blagoderov V, Kitching IJ, Livermore L, Simonsen TJ, Smith VS. No specimen left behind: industrial scale digitization of natural history collections. Zookeys. 2012;(209):133–46. doi: 10.3897/zookeys.209.3178 22859884 PMC3406472

[6] Miralles A, Bruy T, Wolcott K, Scherz MD, Begerow D, Beszteri B, et al. Repositories for Taxonomic Data: Where We Are and What is Missing. Syst Biol. 2020;69(6):1231–53. doi: 10.1093/sysbio/syaa026 32298457 PMC7584136

[7] Allan EL, Livermore L, Price BW, Shchedrina O, Smith VS. A novel automated mass digitisation workflow for natural history microscope slides. Biodivers Data J. 2019;7:e32342. doi: 10.3897/BDJ.7.e32342 PMC6408422 30863197

[8] Hollister JD, Martin G, Cai X, Horton T, Powell O, Sterling M, et al. A Computer Vision Method for Finding Mislabelled Specimens Within Natural History Collections. Ecol Evol. 2025;15(7):e71648. doi: 10.1002/ece3.71648 40655453 PMC12256128

[9] Löbl I, Klausnitzer B, Hartmann M, Krell F-T. The Silent Extinction of Species and Taxonomists—An Appeal to Science Policymakers and Legislators. Diversity. 2023;15(10):1053. doi: 10.3390/d15101053

[10] Engel MS, Ceríaco LMP, Daniel GM, Dellapé PM, Löbl I, Marinov M. The taxonomic impediment: a shortage of taxonomists, not the lack of technical approaches. Zool J Linn Soc. 2021;193:381–7. doi: 10.1093/zoolinnean/zlab072

[11] Gaston KJ, May RM. Taxonomy of taxonomists. Nature. 1992;356(6367):281–2. doi: 10.1038/356281a0

[12] Costello MJ, Wilson S, Houlding B. More taxonomists describing significantly fewer species per unit effort may indicate that most species have been discovered. Syst Biol. 2013;62:616–24. doi: 10.1093/sysbio/syt024 23576317

[13] Bickford D, Lohman DJ, Sodhi NS, Ng PKL, Meier R, Winker K, et al. Cryptic species as a window on diversity and conservation. Trends Ecol Evol. 2007;22(3):148–55. doi: 10.1016/j.tree.2006.11.004 17129636

[14] Pinho C, Kaliontzopoulou A, Ferreira CA, Gama J. Identification of morphologically cryptic species with computer vision models: wall lizards (Squamata: Lacertidae: Podarcis) as a case study. Zool J Linn Soc. 2023;198:184–201. doi: 10.1093/zoolinnean/zlac087

[15] Reginato LF, Brandl SJ. Integrating deep learning, biological hierarchies, and high-resolution imagery to create a new identification tool for cryptic coral reef fishes. PLoS One. 2026;21(6):e0349646. doi: 10.1371/journal.pone.0349646 42241402 PMC13235906

[16] Hollister JD, Paz-García DA, Beas-Luna R, Horton T, Cai X, Fenberg PB. Genes, shells, and AI: using computer vision to detect cryptic morphological divergence between genetically distinct populations of limpets. Sci Rep. 2025;16(1):1051. doi: 10.1038/s41598-025-30613-1 41388058 PMC12783718

[17] Hollister JD, Cai X, Horton T, Price BW, Zarzyczny KM, Fenberg PB. Using computer vision to identify limpets from their shells: a case study using four species from the Baja California peninsula. Front Mar Sci. 2023;10. doi: 10.3389/fmars.2023.1167818

[18] MacLeod N, Canty RJ, Polaszek A. Morphology-Based Identification of *Bemisia tabaci* Cryptic Species Puparia via Embedded Group-Contrast Convolution Neural Network Analysis. Syst Biol. 2022;71(5):1095–109. doi: 10.1093/sysbio/syab098 34951634 PMC9366445

[19] Couret J, Moreira DC, Bernier D, Loberti AM, Dotson EM, Alvarez M. Delimiting cryptic morphological variation among human malaria vector species using convolutional neural networks. PLoS Negl Trop Dis. 2020;14(12):e0008904. doi: 10.1371/journal.pntd.0008904 33332415 PMC7745989

[20] Thevenoux R, LE VL, Villessèche H, Buisson A, Beurton-Aimar M, Grenier E, et al. Image based species identification of Globodera quarantine nematodes using computer vision and deep learning. Computers and Electronics in Agriculture. 2021;186:106058. doi: 10.1016/j.compag.2021.106058

[21] Lu J, Zhang S, Zhao S, Li D, Zhao R. A Metric-Based Few-Shot Learning Method for Fish Species Identification with Limited Samples. Animals (Basel). 2024;14(5):755. doi: 10.3390/ani14050755 38473140 PMC11487373

[22] Figueroa-Mata G, Mata-Montero E. Using a Convolutional Siamese Network for Image-Based Plant Species Identification with Small Datasets. Biomimetics (Basel). 2020;5(1):8. doi: 10.3390/biomimetics5010008 32121572 PMC7148474

[23] Abeysinghe C, Welivita A, Perera I. Snake Image Classification using Siamese Networks. In: Proceedings of the 2019 3rd International Conference on Graphics and Signal Processing, 2019. 8–12. 10.1145/3338472.3338476

[24] Boeters HD, Glöer P, Slavevska Stamenković V. The *Radomaniola/Grossuana* group from the Balkan Peninsula, with a description of *Grossuana maceradica* n. sp. and the designation of a neotype of *Paludina hohenackeri* Küster, 1853 (Caenogastropoda: Truncatelloidea: Hydrobiidae). archmoll. 2017;146(2):187–202. doi: 10.1127/arch.moll/146/187-202

[25] Delicado D, Hauffe T. Shell features and anatomy of the springsnail genus Radomaniola (Caenogastropoda: Hydrobiidae) show a different pace and mode of evolution over five million years. Zoological Journal of the Linnean Society. 2022;196(1):393–441. doi: 10.1093/zoolinnean/zlab121

[26] Glöer P. The Freshwater Gastropods of the West-Palaearctic, Volume 3 Hydrobiidae, Identification Key, Anatomy, Ecology, Distribution. Hetlingen, Germany: Biodiversity Research Lab, 2022.

[27] Geirhos R, Jacobsen JH, Michaelis C, Zemel R, Brendel W, Bethge M. Shortcut learning in deep neural networks. Nature Machine Intelligence. 2020;2:665–73. doi: 10.1038/s42256-020-00257-z

[28] Wilke T, Pfenninger M, Davis GM. Anatomical variation in cryptic mudsnail species: Statistical discrimination and evolutionary significance. Annal N Y Acad Sci. 2002;152:45–66. doi: 10.1635/0097-3157(2002)152[0045:AVICMS]2.0.CO;2

[29] Delicado D, Boulaassafer K, Khalloufi N, Hauffe T. A holistic perspective on species delimitation outperforms all methods based on single data types in freshwater gastropods (Caenogastropoda: Hydrobiidae: *Pseudamnicola*). Zool J Linn Soc. 2025;203:zlae010. doi: 10.1093/zoolinnean/zlae010

[30] International Union for Conservation of Nature IUCN. Habitats Classification Scheme (Version 3.1). IUCN Red List of Threatened Species. https://www.iucnredlist.org/resources/habitat-classification-scheme 2012. Accessed 2024 May 23.

[31] WorldClim Database. https://www.worldclim.org/ Accessed 2024 May 23.

[32] Asch K. IGME 5000: 1: 5 million international geological map of Europe and adjacent areas. Hannover: BGR. 2005.

[33] R Core T. R: A Language and Environment for Statistical Computing. Vienna, Austria: R Foundation for Statistical Computing. 2023.

[34] Hijmans RJ. Terra: Spatial Data Analysis. 2023. doi: 10.32614/CRAN.package.terra

[35] Massicotte P, South A. rnaturalearth: World Map Data from Natural Earth. 2023. doi: 10.32614/CRAN.package.rnaturalearth

[36] Ravi N, Gabeur V, Hu YT, Hu R, Ryali C, Ma T. SAM 2: Segment Anything in Images and Videos. 2024. doi: 10.48550/arXiv.2408.00714

[37] Oquab M, Bottou L, Laptev I, Sivic J. Learning and Transferring Mid-level Image Representations Using Convolutional Neural Networks. In: 2014 IEEE Conference on Computer Vision and Pattern Recognition, 2014. 1717–24. 10.1109/cvpr.2014.222

[38] Pan SJ, Yang Q. A Survey on Transfer Learning. IEEE Trans Knowl Data Eng. 2010;22(10):1345–59. doi: 10.1109/tkde.2009.191

[39] Caruana R. Multitask Learning. Machine Learning. 1997;28:41–75. doi: 10.1023/A:1007379606734

[40] Thrun S. Is learning the n-th thing any easier than learning the first?. Advances in Neural Information Processing Systems. MIT Press. 1995.

[41] Kendall A, Gal Y, Cipolla R. Multi-Task Learning Using Uncertainty to Weigh Losses for Scene Geometry and Semantics. arXiv. 2018. http://arxiv.org/abs/1705.07115

[42] Zhang Y, Yang Q. An overview of multi-task learning. National Science Review. 2017;5(1):30–43. doi: 10.1093/nsr/nwx105

[43] Deng J, Dong W, Socher R, Li L-J, Kai Li, Li Fei-Fei. ImageNet: A large-scale hierarchical image database. In: 2009 IEEE Conference on Computer Vision and Pattern Recognition, 2009. 248–55. 10.1109/cvpr.2009.5206848

[44] Ngiam J, Khosla A, Kim M, Nam J, Lee H, Ng AY. Multimodal deep learning. In: Proceedings of the 28th international conference on machine learning (ICML-11), 2011. 689–96.

[45] Baltrušaitis T, Ahuja C, Morency LP. Multimodal Machine Learning: A Survey and Taxonomy. arXiv. 2017. http://arxiv.org/abs/1705.09406 10.1109/TPAMI.2018.2798607 29994351

[46] Gao J, Li P, Chen Z, Zhang J. A Survey on Deep Learning for Multimodal Data Fusion. Neural Comput. 2020;32(5):829–64. doi: 10.1162/neco_a_01273 32186998

[47] Mroueh Y, Marcheret E, Goel V. Deep multimodal learning for Audio-Visual Speech Recognition. In: 2015 IEEE International Conference on Acoustics, Speech and Signal Processing (ICASSP), 2015. 2130–4. 10.1109/icassp.2015.7178347

[48] Ouyang W, Chu X, Wang X. Multi-source Deep Learning for Human Pose Estimation. In: 2014 IEEE Conference on Computer Vision and Pattern Recognition, 2014. 2337–44. 10.1109/cvpr.2014.299

[49] Nguyen T-L, Kavuri S, Lee M. A multimodal convolutional neuro-fuzzy network for emotion understanding of movie clips. Neural Netw. 2019;118:208–19. doi: 10.1016/j.neunet.2019.06.010 31299625

[50] Hong C, Yu J, Wan J, Tao D, Wang M. Multimodal Deep Autoencoder for Human Pose Recovery. IEEE Trans Image Process. 2015;24(12):5659–70. doi: 10.1109/TIP.2015.2487860 26452284

[51] Koch G, Zemel R, Salakhutdinov R. Siamese Neural Networks for One-shot Image Recognition. In: 2015.

[52] Hoffer E, Ailon N. Deep Metric Learning Using Triplet Network. Lecture Notes in Computer Science. Springer International Publishing. 2015. p. 84–92. doi: 10.1007/978-3-319-24261-3_7

[53] Chopra S, Hadsell R, LeCun Y. Learning a Similarity Metric Discriminatively, with Application to Face Verification. In: 2005 IEEE Computer Society Conference on Computer Vision and Pattern Recognition (CVPR’05). 539–46. 10.1109/cvpr.2005.202

[54] Baldi P, Chauvin Y. Neural Networks for Fingerprint Recognition. Neural Computation. 1993;5(3):402–18. doi: 10.1162/neco.1993.5.3.402

[55] Bromley J, Guyon I, LeCun Y, Säckinger E, Shah R. Signature verification using a “siamese” time delay neural network. In: Advances in Neural Information Processing Systems, 1993. https://proceedings.neurips.cc/paper/1993/hash/288cc0ff022877bd3df94bc9360b9c5d-Abstract.html

[56] Schroff F, Kalenichenko D, Philbin J. FaceNet: A Unified Embedding for Face Recognition and Clustering. In: 2015 IEEE Conference on Computer Vision and Pattern Recognition (CVPR), 2015. 815–23. 10.1109/CVPR.2015.7298682

[57] Xuan H, Stylianou A, Pless R. Improved Embeddings with Easy Positive Triplet Mining. In: 2020 IEEE Winter Conference on Applications of Computer Vision (WACV), 2020. 2463–71. 10.1109/wacv45572.2020.9093432

[58] Qian Q, Shang L, Sun B, Hu J, Tacoma T, Li H. SoftTriple Loss: Deep Metric Learning Without Triplet Sampling. In: 2019 IEEE/CVF International Conference on Computer Vision (ICCV), 2019. 6449–57. 10.1109/ICCV.2019.00655

[59] Deng J, Guo J, Yang J, Xue N, Kotsia I, Zafeiriou S. ArcFace: Additive Angular Margin Loss for Deep Face Recognition. IEEE Trans Pattern Anal Mach Intell. 2022;44(10):5962–79. doi: 10.1109/TPAMI.2021.3087709 34106845

[60] Wu C-Y, Manmatha R, Smola AJ, Krahenbuhl P. Sampling Matters in Deep Embedding Learning. In: 2017 IEEE International Conference on Computer Vision (ICCV), 2017. 2859–67. 10.1109/iccv.2017.309

[61] Musgrave K, Belongie S, Lim S-N. A Metric Learning Reality Check. Lecture Notes in Computer Science. Springer International Publishing. 2020. p. 681–99. doi: 10.1007/978-3-030-58595-2_41

[62] Chen T, Kornblith S, Norouzi M, Hinton G. A simple framework for contrastive learning of visual representations. 2020. doi: 10.48550/arXiv.2002.05709

[63] Grill JB, Strub F, Altché F, Tallec C, Richemond P, Buchatskaya E, et al. Bootstrap Your Own Latent - A New Approach to Self-Supervised Learning. In: Advances in Neural Information Processing Systems, 2020. 21271–84. https://proceedings.neurips.cc/paper/2020/hash/f3ada80d5c4ee70142b17b8192b2958e-Abstract.html

[64] Oord Avd, Li Y, Vinyals O. Representation Learning with Contrastive Predictive Coding. arXiv; 2019. 10.48550/arXiv.1807.03748

[65] Caron M, Touvron H, Misra I, Jegou H, Mairal J, Bojanowski P, et al. Emerging Properties in Self-Supervised Vision Transformers. In: 2021 IEEE/CVF International Conference on Computer Vision (ICCV), 2021. 9630–40. 10.1109/iccv48922.2021.00951

[66] He K, Fan H, Wu Y, Xie S, Girshick R. Momentum Contrast for Unsupervised Visual Representation Learning. In: 2020 IEEE/CVF Conference on Computer Vision and Pattern Recognition (CVPR), 2020. 9726–35. 10.1109/cvpr42600.2020.00975

[67] Gui J, Chen T, Zhang J, Cao Q, Sun Z, Luo H. A Survey on Self-supervised Learning: Algorithms, Applications, and Future Trends. arXiv. 2024. doi: 10.48550/arXiv.2301.05712 38885108

[68] Vaze S, Hant K, Vedaldi A, Zisserman A. Generalized Category Discovery. In: 2022 IEEE/CVF Conference on Computer Vision and Pattern Recognition (CVPR), 2022. 7482–91. 10.1109/cvpr52688.2022.00734

[69] Zhao B, Wen X, Han K. Learning Semi-supervised Gaussian Mixture Models for Generalized Category Discovery. In: 2023 IEEE/CVF International Conference on Computer Vision (ICCV), 2023. 16577–87. 10.1109/iccv51070.2023.01524

[70] Choi S, Kang D, Cho M. Contrastive Mean-Shift Learning for Generalized Category Discovery. In: 2024 IEEE/CVF Conference on Computer Vision and Pattern Recognition (CVPR), 2024. 23094–104. 10.1109/cvpr52733.2024.02179

[71] Howard A, Sandler M, Chen B, Wang W, Chen L-C, Tan M, et al. Searching for MobileNetV3. In: 2019 IEEE/CVF International Conference on Computer Vision (ICCV), 2019. 1314–24. 10.1109/iccv.2019.00140

[72] Hadsell R, Chopra S, LeCun Y. Dimensionality Reduction by Learning an Invariant Mapping. In: 2006 IEEE Computer Society Conference on Computer Vision and Pattern Recognition - Volume 2 (CVPR’06), 2006. 1735–42. 10.1109/CVPR.2006.100

[73] Luo H, Gu Y, Liao X, Lai S, Jiang W. Bag of Tricks and a Strong Baseline for Deep Person Re-Identification. In: 2019 IEEE/CVF Conference on Computer Vision and Pattern Recognition Workshops (CVPRW), 2019. 1487–95. 10.1109/CVPRW.2019.00190

[74] Loshchilov I, Hutter F. SGDR: Stochastic Gradient Descent with Warm Restarts. In: 2017. http://arxiv.org/abs/1608.03983

[75] Bardes A, Ponce J, LeCun Y. VICReg: Variance-Invariance-Covariance Regularization for Self-Supervised Learning. arXiv. 2022. 10.48550/arXiv.2105.04906

[76] Zhai A, Wu HY. Classification is a strong baseline for deep metric learning. In: 2019. 10.48550/arXiv.1811.12649

[77] Wang H, Wang Y, Zhou Z, Ji X, Gong D, Zhou J. CosFace: Large Margin Cosine Loss for Deep Face Recognition. In: 2018. 10.48550/arXiv.1801.09414

[78] Musgrave K, Belongie S, Lim SN. PyTorch Metric Learning. arXiv. 2020. 10.48550/arXiv.2008.09164

[79] Holm S. A Simple Sequentially Rejective Multiple Test Procedure. Scandinavian Journal of Statistics. 1979;6:65–70.

[80] Van Der Maaten L, Hinton G. Visualizing Data using t-SNE. Journal of Machine Learning Research. 2008;9:2579–605.

[81] Guo C, Pleiss G, Sun Y, Weinberger KQ. On Calibration of Modern Neural Networks. arXiv. 2017. doi: 10.48550/arXiv.1706.04599

[82] Youden WJ. Index for rating diagnostic tests. Cancer. 1950;3(1):32–5. doi: 10.1002/1097-0142(1950)3:1<32::aid-cncr2820030106>3.0.co;2-3 15405679

[83] Selvaraju RR, Cogswell M, Das A, Vedantam R, Parikh D, Batra D. Grad-CAM: Visual Explanations from Deep Networks via Gradient-Based Localization. In: 2017 IEEE International Conference on Computer Vision (ICCV), 2017. 618–26. 10.1109/iccv.2017.74

[84] Pantazis O, Brostow GJ, Jones KE, Aodha OM. Focus on the Positives: Self-Supervised Learning for Biodiversity Monitoring. In: 2021 IEEE/CVF International Conference on Computer Vision (ICCV), 2021. 10563–72. 10.1109/iccv48922.2021.01041

[85] Tarasiou M, Zafeiriou S. Embedding Earth: Self-supervised contrastive pre-training for dense land cover classification. 2022. doi: 10.48550/arXiv.2203.06041

[86] Gong Z, Wang AT, Huo X, Haurum JB, Lowe SC, Taylor GW. CLIBD: Bridging Vision and Genomics for Biodiversity Monitoring at Scale. In: arXiv.org, 2024. https://arxiv.org/abs/2405.17537v5

[87] Stevens S, Wu J, Thompson MJ, Campolongo EG, Song CH, Carlyn DE, et al. BioCLIP: A Vision Foundation Model for the Tree of Life. In: 2024 IEEE/CVF Conference on Computer Vision and Pattern Recognition (CVPR), 2024. 19412–24. 10.1109/cvpr52733.2024.01836

[88] Gu J, Stevens S, Campolongo E, Thompson M, Zhang N, Wu J, et al. BioCLIP 2: Emergent Properties from Scaling Hierarchical Contrastive Learning. In: Advances in Neural Information Processing Systems 38, 2025. 113880–913. 10.52202/085713-3436

[89] Gharaee Z, Lowe S, Gong Z, Arias P, Pellegrino N, Wang A, et al. BIOSCAN-5M: A Multimodal Dataset for Insect Biodiversity. In: Advances in Neural Information Processing Systems 37, 2024. 36285–313. 10.52202/079017-1144

[90] Koh PW, Sagawa S, Marklund H, Xie SM, Zhang M, Balsubramani A, et al. WILDS: A Benchmark of In-the-Wild Distribution Shifts. In: Proceedings of the 38th International Conference on Machine Learning, 2021. 5637–64. https://proceedings.mlr.press/v139/koh21a.html

[91] Beery S, Van Horn G, Perona P. Recognition in Terra Incognita. Lecture Notes in Computer Science. Springer International Publishing. 2018. p. 472–89. doi: 10.1007/978-3-030-01270-0_28

[92] Schneider S, Greenberg S, Taylor GW, Kremer SC. Three critical factors affecting automated image species recognition performance for camera traps. Ecol Evol. 2020;10(7):3503–17. doi: 10.1002/ece3.6147 32274005 PMC7141055

[93] Bodesheim P, Blunk J, Körschens M, Brust CA, Käding C, Denzler J. Pre-trained models are not enough: active and lifelong learning is important for long-term visual monitoring of mammals in biodiversity research—Individual identification and attribute prediction with image features from deep neural networks and decoupled decision models applied to elephants and great apes. Mamm Biol. 2022;102:875–97. doi: 10.1007/s42991-022-00224-8

[94] Velasco-Montero D, Fernández-Berni J, Carmona-Galán R, Sanglas A, Palomares F. Reliable and efficient integration of AI into camera traps for smart wildlife monitoring based on continual learning. Ecological Informatics. 2024;83:102815. doi: 10.1016/j.ecoinf.2024.102815

[95] Mou C, Liang A, Hu C, Meng F, Han B, Xu F. Monitoring Endangered and Rare Wildlife in the Field: A Foundation Deep Learning Model Integrating Human Knowledge for Incremental Recognition with Few Data and Low Cost. Animals (Basel). 2023;13(20):3168. doi: 10.3390/ani13203168 37893892 PMC10603653

[96] MacLeod N, Benfield M, Culverhouse P. Time to automate identification. Nature. 2010;467(7312):154–5. doi: 10.1038/467154a 20829777

[97] Padial JM, Miralles A, De la Riva I, Vences M. The integrative future of taxonomy. Front Zool. 2010;7:16. doi: 10.1186/1742-9994-7-16 20500846 PMC2890416

[98] Dayrat B. Towards integrative taxonomy. Biol J Linn Soc. 2005;85:407–17. doi: 10.1111/j.1095-8312.2005.00503.x

[99] Khosla P, Teterwak P, Wang C, Sarna A, Tian Y, Isola P. Supervised Contrastive Learning. 2020. doi: 10.48550/ARXIV.2004.11362

