# Supplementary material for: Speeding up taxonomy in the digital age: A deep learning approach for identifying cryptic freshwater snails
Source: PLoS Comput Biol. 2026 Sep 2;22(9):e1014733. doi: 10.1371/journal.pcbi.1014733 (PMC13561421; doi:10.1371/journal.pcbi.1014733)
Supplement: Supplementary file 1 [file pcbi.1014733.s002.docx]

# Response to Reviewers

Dear Editor,

We thank you for the consideration of our manuscript “Speeding up taxonomy in the digital age: A deep learning approach for identifying cryptic freshwater snails” (Manuscript Number: PCOMPBIOL-D-26-00450) and we would like to thank the reviewers for their thorough evaluation of the manuscript and for the insightful and constructive comments provided. We have carefully considered all suggestions and revised the manuscript accordingly. Below, we present a detailed, point‑by‑point response (in italics) explaining how each comment has been addressed, with all revisions clearly indicated using 'Tracked Changes' in the revised manuscript, in accordance with the journal's submission guidelines.

# Reviewer 1

**Comment #1.** It is recommended to provide comprehensive hyperparameters of the DNN architecture (Fig. 4), such as the number of nodes and layers in the MLP, dropout rate, and the number of input features (especially shell measurements and collection site metadata).

*We revised Fig 4 to include BatchNorm layers, activation functions, dropout parameters, as well as the number of nodes in the layer:*

***Fig 4: Architecture of the classification system.*** *A pre-trained CNN extracts image features from the input images. The image features and the upscaled measurements are concatenated to create a joint representation, which is mapped to an embedding used for classification. Genetic information is incorporated only during training to inform the joint optimization of embeddings and classification results.*

**Comment #2.** It is recommended to provide a brief description for each shell measurement and collection site metadata included in the model. Additionally, clarify whether numerical variables were scaled and how text variables were encoded.

*We added additional information to our exploration of spurious correlations:
The full set of features used, together with their value range and how they were pre-processed is included in Table 1.*

***Table 1. Shell measurements and collection site metadata with values and preprocessing.***

| *Feature* | *Values* | *Preprocessing* |
| --- | --- | --- |
| *Aperture length [mm]* | *0.60-1.53 (1.00 ± 0.15)* | *BatchNorm* |
| *Aperture width [mm]* | *0.22-1.30 (0.82 ± 0.13)* | *BatchNorm* |
| *Shell length [mm]* | *1.30-3.33 (2.09 ± 0.44)* | *BatchNorm* |
| *Shell width [mm]* | *0.81-2.10 (1.42 ± 0.23)* | *BatchNorm* |
| *Width of body whorl [mm]* | *0.70-2.00 (1.26 ± 0.22)* | *BatchNorm* |
| *Width of penultimate whorl [mm]* | *0.41-1.36 (0.79 ± 0.19)* | *BatchNorm* |
| *Country of collection* | *‘Albania’, ‘Bosnia and Herz.’, ‘Croatia’, ‘Greece’, ‘Montenegro’, or ‘North Macedonia’* | *One-hot encoding* |
| *ecoregion code (FEOW)* | *418, 419, 420, 421, or 424* | *One-hot encoding* |
| *Geological information* | *Three flags: Geol_carb, geol_silic, geol_volc* | *None* |

*Numerical features are listed with value range, mean and standard deviation. Categorical features are listed with the set of possible values. Numerical features were scaled with a learnable BatchNorm layer, categorical features were one-hot encoded.*

**Comment #3.** The description is ambiguous regarding whether the pre-trained CNN used for image feature extraction was fine-tuned during Siamese neural network training or if its weights were frozen (in other words, images were converted into feature vectors in advance before being input into the model).

*We added the following clarification:*

*“During training we only optimize the last layers, namely convolutional block 5, conv head, and the classifier layer that outputs the 128-dimensional image feature vector. All other parameters, including convolutional blocks 0-4 remain fixed to their pre-trained values.”*

**Comment #4.** The model was trained for 20 epochs without early stopping. Given the small size of the training set, overfitting is likely to occur after approximately 1-2 epochs.

*We thank the reviewer for highlighting this potential issue. We adapted the training loop to mitigate this issue:*

*“In each of our experiments, we split the data into three disjoint sets: training, validation, and testing. After each training epoch, the model performance was evaluated against the validation data, and after training, the model with the best performance on the validation data was evaluated against the test set. This also helps mitigate potential overfitting, where the model still improves performance on the training set, but fails to generalize to different data. We used 5-fold cross-validation stratified by class for data splitting and repeated it five times with different splits to obtain more robust results.”*

**Comment #5.** Considering the class imbalance, it is recommended to report the accuracy for each species and further use other evaluation metrics such as precision and recall. Special attention should be paid to whether the accuracy is lower for species with smaller training sample sizes.

*We agree with the reviewer on this point. We changed our performance reporting across all experiments to include the macro-averaged F1 score. F1 score combines precision and recall, providing a more complete insight into the model’s performance, and macro averaging computes separate F1 scores for each class and then computes the unweighted mean of the class scores. With these changes, low precision and/or recall, especially for underrepresented species, is not hidden by a high accuracy for species with a large number of samples.*

*We also added Fig 6, where we investigated class-wise model performance and observed that the variance is much higher for small classes but stable at a high level for classes with more than 20 specimens:*

***Fig 6: Class-wise test-set classification performance for different model configurations.*** *Variance was very high for classes with few samples, spanning from 0 to 1. On the larger classes the models have a consistently good performance.*

**Comment #6.** For the dynamic margin, it is suggested to clarify how genetic distances are mapped to the margin.

*We added the following clarification: “Examples, from our matrix of cophenetic distances include R. bosniaca and R. nachtigallae, which have a distance of 2.02, and R. bosniaca and R. wolffi, with a distance of 6.37. The margin between the classes is then a base margin multiplied by the cophenetic distance. Therefore, the margin between R. bosniaca and R. wolffi is approximately three times as large as the margin between R.bosniaca and R. nachtigallae.”*

**Comment #7.** For users, it would be helpful to know the confidence of prediction. It is recommended to provide a probability threshold or a dedicated uncertainty estimation method. Furthermore, what results may imply a novel species not present in the training set?

*We thank the reviewer for this suggestion. We added an additional in-depth analysis of how the model behaves for novel species (Section “Performance on unseen species”). We also added temperature scaling (Guo et al., 2017) so that the output softmax probabilities more closely represented the actual probability of the model being correct. Two main findings were obtained: (1) new species also formed distinct clusters in the embedding space, similar to known species from new collection sites (Fig 13); (2) with self-supervised training, it was possible to obtain a well-calibrated model, where low-confidence predictions were almost always wrong, and high-confidence predictions were almost always right (Fig 14).*

*One caveat remains: due to the organisation of* Radomaniola *at the location level, it is not possible for our model (and very difficult for trained experts) to differentiate between a new phenotype of a known species and a new species.*

**Comment #8.** When discussing "in-distribution" and "out-of-distribution" data, please provide definitions within the context to ensure clarity.

*We removed references to “in-distribution” and “out-of-distribution”. Instead, we now explicitly refer to testing on specimens from collection sites seen during training (previously “in-distribution”), or collection sites unseen during training (previously “out-of-distribution”).*

**Comment #9.** For metadata not seen during training (e.g., samples collected from other countries), how should they be input into the model?

*Currently, the model cannot handle such cases. We added this to the discussion of the limitations: “In addition, our approach of one-hot encoding categorical variables, such as country of origin, cannot handle metadata unseen during training. Including specimens from a country absent from the training set would require retraining with re-encoded features.”*

# Reviewer 2

**Comment #1.** There are several approaches that could be tried in order to identify the causes of this drop in performance and possibly remedy it, making the pipeline even more useful to taxonomists who would like to apply it to their own data.

Run an ablation study to see performance differences using partial data

Eg dropout collection site data, shell morphology data etc as blocks randomly

Also, random dropout

*We thank the reviewer for this suggestion. Random dropout is already used throughout the network. However, we also added a dropout variant where either all image features, or the measurement features are dropped completely to force the network to learn from both inputs.*

Regularization of features with OSCAR or other penalties on image features

*We thank the reviewer for this suggestion. We added Variance-Invariance-Covariance regularization (Bardes et al, 2022) to regularize embeddings and upscaled the measurement features to avoid the image features dominating.*

Show activation on images

*We added a figure investigating model activations for three cases: (1) correct prediction, true class, (2) incorrect prediction, predicted class, (3) incorrect prediction, true class (Fig 15). Unfortunately, we found that, for the samples we tested, the activation maps do not provide a useful heuristic to detect wrong predictions.*

If regularization and dropout fail to improve the performance when testing on out of distribution data there is another option available:

Test time training techniques: <https://arxiv.org/abs/1909.13231>

*We thank the reviewer for this suggestion. We did not implement test time training, but instead used self-supervised embedding learning, as this aligns closer with our framework. Self-supervised embeddings are learned without class labels and therefore allow the inclusion of unlabeled test data in the training process. We found that this improves f1 score on data from collection sites seen during training, and top-3 accuracy on data from collection sites not seen during training.*

**Comment #2.** Some of the discussion alludes to possible sources of this overfitting

522 - 529 this clustering at the geography level might not be such a big problem for your taxa but others might have to deal with wider geographic spreads

This approach is probably generalizable and worth making available to other taxonomists as a pipeline/ tool.

But first it this possible source of overfitting should prob be tracked down.

*We would like to reiterate that, in our opinion, this drop in performance is not necessarily a model failure and instead captures a valid biological signal; in* Radomaniola *much of the morphometric variation is not organized at the species level, but at the location level. Therefore, specimens from different localities exhibit vastly different morphometric features, which the model is not able to correctly identify without supervision. Nonetheless, once additional labeled data from a new collection site is included in the training, the model is quickly able to adapt to this new locality (See section “Performance with incrementally available labels”, Fig 15).*

534 - 542 It’s good to see that the utility is still there and it could easily be used in the field even in its present state. Maybe test time training can help see as relatively few samples were needed and the predictions were already leaning in the right direction

*We agree. Inclusion of unsupervised training and inclusion of unlabeled test data improved performance in this setting as well.*

**Comment #3.** In addition, I might suggest one extra experiment to be sure there isn’t any leakage of information that can be used/help to infer classes: Can you build a classifier to try to figure out which camera/data acquisition run the pictures were taken with if multiple cameras were used during sampling? This would require running the same pipeline to prep the images but outputting class labels for the camera using only the image net. This might be adding some signal even though its invisible to the naked eye if these camera labels correspond to only one part of the taxa during the training/ testing of in distribution data

*All images were taken with the same camera system, a Keyence VHX 2000 digital microscope in combination with the VHX-2000 communication software v. 2.3.5.0 (Keyence Corporation); we also added this to our methodology. However, there is a high probability that conditions on one site are consistent and conditions vary between collection sites. We tried to remove shortcuts with the removal of the background and the variation of the image color profile (slight changes to contrast, saturation). Even though that might add signal to the images, in our opinion the intra-species differences across different collection sites far dominate, and as indicated by Figures 5, 13, and 16 the model is already able to very efficiently group together specimens from the same collection site.*

**Comment #4.** Another interesting experiment that could be run is visualizing the activations of the network over the input images ( https://github.com/jacobgil/pytorch-grad-cam ) which can help identify which features it is using to make predictions.

*We added a figure showing model activations for three cases: (1) correct prediction, true class, (2) incorrect prediction, predicted class, (3) incorrect prediction, true class (Fig 14). Unfortunately, we found that, for the samples we tested, the activation maps do not provide a reliable heuristic to detect wrong predictions.*

***Fig 15: GradCAM visualization of image regions relevant to the prediction.*** *We compare three cases (1) the model makes the correct prediction, (2) the model makes an incorrect prediction, (3) the relevant regions for the actual class if the model makes an incorrect prediction. All models focus on relevant parts of the shell, inspecting activations does not provide a clear heuristic for recognizing incorrect predictions.*

**Comment #5.** The code for running / training this approach could benefit from a few cli utilities and some documentation to help a user prepare data in similar formats and then apply this approach to creating their own taxonomic classifiers. This might be beyond the scope of this manuscript but I could imagine this approach could be of general utility for teams collecting imaging data with similar metadata.

*We thank the reviewer for this suggestion. We improved documentation in the README.md file and added an additional notebook demonstrating how to use the code for training different models and visualizing model performance, embedding space, confidence, and GradCAM explanations.*

# Reviewer 3

**Comment #1.** Although this is a unique approach to identifying cryptic snails, there are other papers that have been published recently that use CV methods for the same purpose and taxonomic group (molluscs). These should be discussed in the intro and/or discussion as appropriate because they have the same goals but use a different approach. The discussion in general is very lacking in citations (only 2!). For a paper like this, I would expect many more. How else is the reader supposed to put the results of your paper into the wider literature context if you have not cited anything in the discussion? So at the very least, you need to have a discussion around important papers that were published in this field (and taxonomic group) using CV methods:

Hollister, J.D., Paz-García, D.A., Beas-Luna, R., Horton, T., Cai, X. and Fenberg, P.B., 2025. Genes, shells, and AI: using computer vision to detect cryptic morphological divergence between genetically distinct populations of limpets. Scientific Reports.

Hollister, J.D., Cai, X., Horton, T., Price, B.W., Zarzyczny, K.M. and Fenberg, P.B., 2023. Using computer vision to identify limpets from their shells: a case study using four species from the Baja California peninsula. Frontiers in Marine Science, 10, p.1167818.

Line 70: there is an important paper to cite here as well by the same author as above that covers this topic:

Hollister, J.D., Martin, G., Cai, X., Horton, T., Powell, O., Sterling, M., Turnbull, G., Price, B.W. and Fenberg, P.B., 2025. A computer vision method for finding mislabelled specimens within natural history collections. Ecology and Evolution, 15(7), p.e71648.

*We thank the reviewer for raising this valid criticism. We restructured the discussion to better position our results in the wider context, and to show where our approach mirrors and diverges from existing work in the field. We also added appropriate references to all sections of the discussion.*

**Comment #2.** While the methods are generally well described in terms of the model, there are some aspects of the dataset that are not clear. For example, the reader has to find their way to the Zenodo dataset to know what shell measurements are used for each specimen. All you say is that you used “shell measurements”. But this is very vague and does not tell the reader much. What shell measurements were used and why? Is there a big overlap in shell measurements between species and individuals? Molluscs have a large range in size and shape, so it’s important to give more context. Perhaps a table or SI table would be helpful with this information.

*Regarding the large range in size and shape of mollusks, the degree of morphological overlap among species varies considerably among genera and families, depending on the traits examined and the evolutionary history of the group. Shell dimensions are standard taxonomic characters in Hydrobiidae, but the extent of overlap among species is taxon-specific. In* Radomaniola*, shell morphology alone is often insufficient for reliable species identification, which is precisely one of the motivations for integrating multiple sources of information in our approach.*

*In our section discussing potential spurious correlations and the cleaning of measurement values, we added a table describing the actual features and their value ranges as they are used in model training:*

*“The full set of features used, together with their value range and how they were pre-processed is included in Table 1.*

***Table 1. Shell measurements and collection site metadata with values and preprocessing.***

| *Feature* | *Values* | *Preprocessing* |
| --- | --- | --- |
| *Aperture length [mm]* | *0.60-1.53 (1.00 ± 0.15)* | *BatchNorm* |
| *Aperture width [mm]* | *0.22-1.30 (0.82 ± 0.13)* | *BatchNorm* |
| *Shell length [mm]* | *1.30-3.33 (2.09 ± 0.44)* | *BatchNorm* |
| *Shell width [mm]* | *0.81-2.10 (1.42 ± 0.23)* | *BatchNorm* |
| *Width of body whorl [mm]* | *0.70-2.00 (1.26 ± 0.22)* | *BatchNorm* |
| *Width of penultimate whorl [mm]* | *0.41-1.36 (0.79 ± 0.19)* | *BatchNorm* |
| *Country of collection* | *‘Albania’, ‘Bosnia and Herz.’, ‘Croatia’, ‘Greece’, ‘Montenegro’, or ‘North Macedonia’* | *One-hot encoding* |
| *ecoregion code (FEOW)* | *418, 419, 420, 421, or 424* | *One-hot encoding* |
| *Geological information* | *Three flags: Geol_carb, geol_silic, geol_volc* | *None* |

*Numerical features are listed with value range, mean and standard deviation. Categorical features are listed with the set of possible values. Numerical features were scaled with a learnable BatchNorm layer, categorical features were one-hot encoded.”*

**Comment #3.** How did you train species that only have 5 specimens? Is that not too low of a sample size to accurately use them for training purposes? Seems like you need more justification on how accurate the models are for species where you have low samples sizes.

*We agree with the reviewer that this is a valid concern. We investigated this and added the following observation to the manuscript:*

*“We also observed large variances in the macro-averaged F1 score across all methods. This variability is likely attributed to the limited number of samples available for certain species, resulting in test sets containing only one or two specimens. Consequently, the F1 score is either 0 (incorrect prediction) or 1 (correct prediction). With 20 species in total, missing a single prediction can therefore result in a drop of approx. 0.05. Indeed, when we investigated the distribution of F1 scores by class size (Fig 6), we observed that the smaller classes had a much higher variance, spanning from 0 to 1, whereas the performance had consistently high values for larger classes. Only models incorporating self-supervised embeddings and unlabeled test data consistently achieved reliable performance in classes with very few available samples.*

***Fig 6: Class-wise test-set classification performance for different model configurations.*** *Variance was very high for classes with few samples, spanning from 0 to 1. On the larger classes the models have a consistently good performance.”*

*While the model performance on specimens with low sample size may not be indicative of general model performance, we still think inclusion of specimens with low sample size is useful, as in embedding training they can still serve as negative samples to better define the boundaries of species with more available samples.*

**Comment #4.** Line 416: why would you ever use a specimen in a test dataset if they are also in the training data? The whole point of the training data is so you can test it using un-seen data. This seems like a major flaw but I might be missing something. Related to this, you do not mention anywhere how many (or proportion) specimens are in the training, validation, and test datasets per species. Unless I am missing something fundamental here, I think this is a key piece of information that is not present in the study.

*We thank the reviewer for highlighting this imprecise formulation. In our experiments, the training, validation and test sets are distinct; each specimen is in only one of them. In addition, the split is stratified by species, so that the relative distribution of species in the training, validation and test sets is approximately the same, and it resembles the distribution shown in Fig 2.*

*We added clarification in the text:*

*“Overall, the test dataset for this experiment consisted of 55 specimens from 5 different species and 6 different collection sites, with the species distribution shown in Fig 9.*

***Fig 9: Species distributions for training, validation, and test set.*** *Specimens from five species and six collection sites are selected for testing. The remaining specimens are split into five folds, stratified by species; four folds are used for training, one for validation.*

*Similar to our previous experiment, we split the training data into training and validation with a five times repeated five-fold cross-validation split. The model from the epoch where performance was highest on the validation set was then evaluated on the test dataset.*

**Minor Comment #1.** Line 162: what does “drop substantially” mean in terms of statistics?

*We changed the text to “[…] the accuracy dropped to slightly above 80%, […]”.*

**Minor Comment #2.** Line 169: how many “countries” are there in the dataset?

*There are specimens from 6 different countries in the dataset. We added clarifying information regarding our used shell measurements and collection site metadata, as well as their respective value ranges in Table 1 in the revised manuscript (see our response to Comment #2).*

**Minor Comment #3.** It’s hard to tell if you only have images of specimens or if you have the specimens themselves. So a “specimen” is just an image, not an actual physical specimen?

*We added clarification in the text:*

*“For each specimen we have a high-resolution photograph available (see Fig 1), in combination with a series of morphometric measurements and metadata from the collection site. All images were taken with the same camera system, a Keyence VHX 2000 digital microscope in combination with the VHX-2000 communication software v. 2.3.5.0 (Keyence Corporation). Not all specimens are preserved; for dissected individuals the shell had to be broken to allow examination of the soft parts. For the remaining specimens the shell (and the specimen) is preserved and deposited in the Justus Liebig University Giessen (UGSB) collection.”*

**Minor Comment #4.** Are all images taken at the same angle, distance and orientation (e.g. aperture side up?) if so state this.

*We added additional clarification: “All specimens were photographed aperture side up. However, their orientation, distance to the objective, relative size in the image, and spatial resolution (µm per pixel) differ between images; even though relative size in the image and spatial resolution are approximately consistent across photographs from the same collection site.”*

**Minor Comment #5.** Line 35, add some average performance scores here (F1 scores?)

*We added average performances in a later sentence:*

*“On specimens from collection sites seen during training we achieved a macro-averaged F1 score of 0.93. Even though this dropped as low as 0.14 when evaluating on specimens from previously unsampled localities, it could be rapidly recovered by retraining with 2-3 newly labeled specimens. Additionally, model top-3 accuracy stayed consistently above 80% in all settings.”*

**Minor Comment #6.** Lines 74-88: well written, but you are missing some recent studies that have looked at these issues you raise here, please cite papers by Hollister et al. They use CV to identify cryptic marine snails (limpets), both between and within species.

*We added references to Hollister et al., as well as other matching papers to this section.*

**Minor Comment #7.** Line 116: cite Hollister et al. 2025, where they look at cryptic divergence across space within species using CV methods.

*We added additional references to Hollister et al., as well as other appropriate related works.*

**Minor Comment #8.** Line 162: what does “drop substantially” mean in terms of statistics?

*Duplicate, see response to Minor Comment #1*

**Minor Comment #9.** Line 169: how many “countries” are there in the dataset?

*Duplicate, see response to Minor Comment #2.*

**Minor Comment #10.** It’s hard to tell if you only have images of specimens or if you have the specimens themselves. So a “specimen” is just an image, not an actual physical specimen?

*Duplicate, see response to Minor Comment #3.*

**Minor Comment #11.** Are all images taken at the same angle, distance and orientation (e.g. aperture side up?) if so state this.

*Duplicate, see response to Minor Comment #4.*

**Minor Comment #12.** Line 327: who is Adam?

*In this case, ‘Adam’ refers to the stochastic optimization method by Kingma and Ba (*[*https://arxiv.org/abs/1412.6980*](https://arxiv.org/abs/1412.6980)*). This method is widely used in the training of deep neural networks. We have adapted the text to improve clarity regarding this confusion:*

*“Networks were trained for 20 epochs using a batch size of 64 and the Adam optimization method with decoupled weight decay [43]. As optimizer parameters we used a learning rate of 0.001, betas of 0.9 and 0.999 and weight decay factor of 0.1. Learning rate is decayed with a cosine schedule to 10^-8^ [74].”*

**Minor Comment #13.** Fig 5. The x-axis labels do not quite match with what is written in the caption or in the text. Include the “genetic data” at least on the appropriate area so we know which one you’re referring to. What is exactly the “test set performance” metric…is it an F1 score or similar?

*We revised Fig 5 to improve clarity:*

***Fig 5: Test-set classification performance for different model configurations.*** *The dashed line marks the highest median macro-averaged F1 score. Including shell measurements and collection site metadata alongside images improves system performance, learning embeddings and classification simultaneously further increases performance. Integrating genetic information through the dynamic margin did not yield significant changes; self-supervised learning and the inclusion of unlabeled test data produced the largest improvements.*

**Minor Comment #14.** There is a A LOT of hard to navigate jargon in lines 371-380. Please write this is plain language if possible, or explain a bit more.

*We rewrote the section where we compare the different methods for embedding learning. We also included additional text clarifying the different terms and the differences between the methods:
“Method (1) is the Siamese network approach described earlier. Methods (2)-(4) are variations of triplet networks. In this context a “positive” refers to a different specimen from the same species, while a “negative” refers to a specimen from a different species; “easy” or “hard” refers to how similar the specimens seem. For example, a hard positive is a same-species pair, that nonetheless looks quite different, and a semi-hard negative is a different-species pair that looks fairly similar. Overall, Methods (1) – (4) compare samples to each other, while Methods (5) – (8) compare samples to learned representatives. In addition, methods (4) – (8) have internal learnable parameters, for example a class-specific margin penalty in [60]. These internal parameters were trained jointly with the rest of the network, using the same optimizer and training settings. All methods also have non-learnable hyperparameters, such as the target margins between classes. We did not perform a search for optimal values for these hyperparameters, but instead used the values suggested by [61,78].*

*In addition, we also compare multiple self-supervised methods: (9) self-supervised contrastive learning with the InfoNCE loss introduced in Equation (3) [62,64], (10) BYOL [63], and DINO [65]. All three of these methods work by aligning the embeddings belonging to different views of the same input. Each of these methods is evaluated with and without inclusion of unlabeled training data to investigate if integrating additional unlabeled data improves performance.”*

**Minor Comment #15.** What is the justification for the “top 3 accuracy” box plots in figs 5 and 6? What are the “top 3”, this is not explained in the paper.

*We added an additional sub-section regarding our evaluation logic at the end of the Materials and Methods section:*

*“In our experiments, we measure performance with macro-averaged f1 score, as well as top-3 accuracy on the test dataset. F_1_ score is the harmonic mean of precision and recall, defined as*

$$\begin{aligned} F_{1}=2* \frac{precision\cdot recall}{precision+recall}=\frac{2\cdot TP}{2\cdot TP+FP+FN}\#\left( 3 \right) \end{aligned}$$

*with the number of true positives TP, false positives FP, and false negatives FN. The macro-averaged score is then the arithmetic mean of the per-class scores. Macro-averaged F1 score has a value between 0 and 1. A high score indicates that the system consistently predicts the correct species, even for specimens from underrepresented species. This provides a better performance measure than accuracy, as it gives equivalent weight to both precision and recall. In addition, with macro-averaging low performance on a species with few samples cannot be overshadowed by the high performance of a species with many samples.*

*In addition, we evaluated the top-3 accuracy of our models, which is the fraction of predictions, where the correct species is among the three predictions with the highest confidences. This metric is reflective of a real-world setting, where the system is not used as a replacement for a taxonomist, but instead as a support system that provides a short list of three likely candidates for the taxonomist, so they can restrict their efforts to likely species.”*

**Minor Comment #16.** Line 426-429: so there is a lot of intraspecific variability, did you confirm the correct species using genetic data for all specimens?

*No. We added clarification to the manuscript:*

*“Genetic data were not obtained for every individual specimen. Typically, between one and three specimens per population were DNA-sequenced. However, Radomaniola species rarely co-occur at the same locality. In addition, approximately half of the specimens were dissected and their species identity was independently confirmed using anatomical characters. Therefore, species assignments are considered reliable despite not having genetic data for every specimen.”*

**Minor Comment #17.** Figs 10 and 11 are very hard to interpret. Please re-do it so it;’s more visually appealing.

*We reworked Figs 10 and 11 to reduce clutter and duplicate information (Fig 10 is now called Fig 16):*

***Fig 16: Performance on unseen sites with incrementally added labeled samples.*** *The dashed line marks the highest median accuracy score at each retraining. Inclusion of shell measurements and collection site metadata significantly improves performance. In contrast, the different methods for additional embedding learning only have minimal impact.*

**Minor Comment #18.** The discussion is very poorly cited (only 2 citations!). Even for a methods paper like this one, that is very very few for a discussion. How are we to put your study into a wider context with the literature with so few references to similar studies? Again, you should cite similar papers in the field, e.g. Hollister et al.

*We reworked the discussion; see response to Comment #1.*
