# Supplementary material for: Speeding up taxonomy in the digital age: A deep learning approach for identifying cryptic freshwater snails
Source: PLoS Comput Biol. 2026 Sep 2;22(9):e1014733. doi: 10.1371/journal.pcbi.1014733 (PMC13561421; doi:10.1371/journal.pcbi.1014733)
Supplement: Peer review history

# Peer review: Decision Letter 0

**DOI:** 10.1371/journal.pcbi.1014733.r001

**Authors:** Pedro Mendes, Miguel Francisco de Almeida Pereira de Rocha

4 Jun 2026

PCOMPBIOL-D-26-00450

Speeding up taxonomy in the digital age: A deep learning approach for identifying cryptic freshwater snails

PLOS Computational Biology

Dear Dr. Vetter,

Thank you for submitting your manuscript to PLOS Computational Biology. After careful consideration, we feel that it has merit but does not fully meet PLOS Computational Biology's publication criteria as it currently stands. Therefore, we invite you to submit a revised version of the manuscript that addresses the points raised during the review process.

Please submit your revised manuscript by Aug 04 2026 11:59PM. If you will need more time than this to complete your revisions, please reply to this message or contact the journal office at ploscompbiol@plos.org. When you're ready to submit your revision, log on to https://www.editorialmanager.com/pcompbiol/ and select the 'Submissions Needing Revision' folder to locate your manuscript file.

Please include the following items when submitting your revised manuscript:

* A letter that responds to each point raised by the editor and reviewer(s). You should upload this letter as a separate file labeled 'Response to Reviewers'. This file does not need to include responses to formatting updates and technical items listed in the 'Journal Requirements' section below.

* A marked-up copy of your manuscript that highlights changes made to the original version. You should upload this as a separate file labeled 'Revised Manuscript with Track Changes'.

* An unmarked version of your revised paper without tracked changes. You should upload this as a separate file labeled 'Manuscript'.

If you would like to make changes to your financial disclosure, competing interests statement, or data availability statement, please make these updates within the submission form at the time of resubmission. Guidelines for resubmitting your figure files are available below the reviewer comments at the end of this letter. As the corresponding author, your ORCID iD is verified in the submission system and will appear in the published article. PLOS supports the use of ORCID, and we encourage all coauthors to register for an ORCID iD and use it as well. Please encourage your coauthors to verify their ORCID iD within the submission system before final acceptance, as unverified ORCID iDs will not appear in the published article. Only the individual author can complete the verification step; PLOS staff cannot verify ORCID iDs on behalf of authors.

We look forward to receiving your revised manuscript.

Kind regards,

Miguel Francisco de Almeida Pereira de Rocha

Academic Editor

PLOS Computational Biology

Pedro Mendes

Section Editor

PLOS Computational Biology

**Journal Requirements:**

1) We do not publish any copyright or trademark symbols that usually accompany proprietary names, eg ©,  ®, or TM  (e.g. next to drug or reagent names). Therefore please remove all instances of trademark/copyright symbols throughout the text, including:

- ® on page: 26.

2) Some material included in your submission may be copyrighted. According to PLOS's copyright policy, authors who use figures or other material (e.g., graphics, clipart, maps) from another author or copyright holder must demonstrate or obtain permission to publish this material under the Creative Commons Attribution 4.0 International (CC BY 4.0) License used by PLOS journals. Please closely review the details of PLOS's copyright requirements here: <a data-cke-saved-href="https://journals.plos.org/ploscompbiol/s/licenses-and-copyright" href="https://journals.plos.org/ploscompbiol/s/licenses-and-copyright">PLOS Licenses and Copyright</a>. If you need to request permissions from a copyright holder, you may use the&nbsp;<a data-cke-saved-href="https://storage.googleapis.com/plos-published-prod/c4ff/Content%20Copyright%20Permission%20Form.pdf?X-Goog-Algorithm=GOOG4-RSA-SHA256&amp;X-Goog-Credential=wombat-sa%40plos-prod.iam.gserviceaccount.com%2F20220817%2Fauto%2Fstorage%2Fgoog4_request&amp;X-Goog-Date=20220817T210608Z&amp;X-Goog-Expires=86400&amp;X-Goog-SignedHeaders=host&amp;X-Goog-Signature=cd9dc868773378f7033b9f8cfb91430167d6275df90bce01342a454f36512530b64f24736fbf68c4fd96da74d663b6ee95b3420bded8fb0e833af2390b896b8706643b476d7f532408921b60576d65b60617070900fd2bcd28eec4cd32fefcf007d3655df17fcc91023329a28479ce0d1c5758174eedcc9e3c1788c5f7dd8523f3ddd871e02c998a20f7dc78ec00f313d4b5a78ba2037a6c7ef0a05dc29817da9368065460c8d8446659f35f0458fefabd3101eb7f926e9cb7a0b53e7bbe47aaed3e0ba52f9af9c2b8f6d7d3a8871b205fc4cbe97384ed37c7604fd7eecfedccf49cba12820b2e3b649541cefde3103cd7d72731b3e8b0248439b4cba691ca8a" href="https://storage.googleapis.com/plos-published-prod/c4ff/Content%20Copyright%20Permission%20Form.pdf?X-Goog-Algorithm=GOOG4-RSA-SHA256&amp;X-Goog-Credential=wombat-sa%40plos-prod.iam.gserviceaccount.com%2F20220817%2Fauto%2Fstorage%2Fgoog4_request&amp;X-Goog-Date=20220817T210608Z&amp;X-Goog-Expires=86400&amp;X-Goog-SignedHeaders=host&amp;X-Goog-Signature=cd9dc868773378f7033b9f8cfb91430167d6275df90bce01342a454f36512530b64f24736fbf68c4fd96da74d663b6ee95b3420bded8fb0e833af2390b896b8706643b476d7f532408921b60576d65b60617070900fd2bcd28eec4cd32fefcf007d3655df17fcc91023329a28479ce0d1c5758174eedcc9e3c1788c5f7dd8523f3ddd871e02c998a20f7dc78ec00f313d4b5a78ba2037a6c7ef0a05dc29817da9368065460c8d8446659f35f0458fefabd3101eb7f926e9cb7a0b53e7bbe47aaed3e0ba52f9af9c2b8f6d7d3a8871b205fc4cbe97384ed37c7604fd7eecfedccf49cba12820b2e3b649541cefde3103cd7d72731b3e8b0248439b4cba691ca8a">PLOS Content Copyright Permission&nbsp;Form</a>

Please respond directly to this email and provide any known details concerning your material's license terms and permissions required for reuse, even if you have not yet obtained copyright permissions or are unsure of your material's copyright compatibility. Once you have responded and addressed all other outstanding technical requirements, you may resubmit your manuscript within Editorial Manager.

Potential Copyright Issues:

- Please confirm (a) that you are the photographer of Figures 1, 3, and 4 and 8., or (b) provide written permission from the photographer to publish the photo(s) under our CC BY 4.0 license.

**Reviewers' comments:**

Reviewer's Responses to Questions

**Comments to the Authors:**

**Please note here if the review is uploaded as an attachment.**

Reviewer #1: This manuscript proposes a deep learning approach for species identification of cryptic freshwater snails. The integration of morphology and habitat information is interesting and promising. The Siamese neural network is suitable for scenarios with scarce training samples and class imbalance. However, the manuscript lacks some details regarding the deep learning methodology. Several concerns and suggestions are outlined below.

1. It is recommended to provide comprehensive hyperparameters of the DNN architecture (Fig. 4), such as the number of nodes and layers in the MLP, dropout rate, and the number of input features (especially shell measurements and collection site metadata).

2. It is recommended to provide a brief description for each shell measurement and collection site metadata included in the model. Additionally, clarify whether numerical variables were scaled and how text variables were encoded.

3. The description is ambiguous regarding whether the pre-trained CNN used for image feature extraction was fine-tuned during Siamese neural network training or if its weights were frozen (in other words, images were converted into feature vectors in advance before being input into the model).

4. The model was trained for 20 epochs without early stopping. Given the small size of the training set, overfitting is likely to occur after approximately 1-2 epochs.

5. Considering the class imbalance, it is recommended to report the accuracy for each species and further use other evaluation metrics such as precision and recall. Special attention should be paid to whether the accuracy is lower for species with smaller training sample sizes.

6. For the dynamic margin, it is suggested to clarify how genetic distances are mapped to the margin.

7. For users, it would be helpful to know the confidence of prediction. It is recommended to provide a probability threshold or a dedicated uncertainty estimation method. Furthermore, what results may imply a novel species not present in the training set?

8. When discussing "in-distribution" and "out-of-distribution" data, please provide definitions within the context to ensure clarity.

9. For metadata not seen during training (e.g., samples collected from other countries), how should they be input into the model?

Reviewer #2: I found this paper to be an enjoyable read and a good case study/ example of how to apply machine learning without massive compute budgets and gargantuan datasets.

The use of methods to deal with the limitations of the small imbalance dataset are impressive and appropriate. The paper is a nice refresher on the appropriate use the following techniques.

Transfer learning

Siamese or triplet networks

Contrastive loss

Uncertainty weighting and multitask loss function building

Image augmentation ( with attention to biological detail )

Exhaustive methods evaluation for triplets vs siamese and loss functions

The performance metrics of the network and general good standard practices of the author make the case that this is a tool with real world utility. However, the one weakness that the authors also recognize and describe at length is the performance of the network on out of distribution data.

There are several approaches that could be tried in order to identify the causes of this drop in performance and possibly remedy it, making the pipeline even more useful to taxonomists who would like to apply it to their own data.

Run an ablation study to see performance differences using partial data

Eg dropout collection site data, shell morphology data etc as blocks randomly

Also, random dropout

Regularization of features with OSCAR or other penalties on image features

Show activation on images

If regularization and dropout fail to improve the performance when testing on out of distribution data there is another option available:

Test time training techniques : https://arxiv.org/abs/1909.13231

Some of the discussion alludes to possible sources of this overfitting

522 - 529 this clustering at the geography level might not be such a big problem for your taxa but others might have to deal with wider geographic spreads

This approach is probably generalizable and worth making available to other taxonomists as a pipeline/ tool.

But first it this possible source of overfitting should prob be tracked down.

534 - 542 It’s good to see that the utility is still there and it could easily be used in the field even in its present state. Maybe test time training can help see as

relatively few samples were needed and the predictions were already leaning in the right direction

In addition, I might suggest one extra experiment to be sure there isn’t any leakage of information that can be used/help to infer classes: Can you build a classifier to try to figure out which camera/data acquisition run the pictures were taken with if multiple cameras were used during sampling? This would require running the same pipeline to prep the images but outputting class labels for the camera using only the image net. This might be adding some signal even though its invisible to the naked eye if these camera labels correspond to only one part of the taxa during the training/ testing of in distribution data

Another interesting experiment that could be run is visualizing the activations of the network over the input images ( https://github.com/jacobgil/pytorch-grad-cam ) which can help identify which features it is using to make predictions.

All in all I find the pipeline and approach to be appropriate to the problem and the results convincing. The code for running / training this approach could benefit from a few cli utilities and some documentation to help a user prepare data in similar formats and then apply this approach to creating their own taxonomic classifiers. This might be beyond the scope of this manuscript but I could imagine this approach could be of general utility for teams collecting imaging data with similar metadata.

Reviewer #3: Dear authors,

I quite enjoyed reading your paper on using CV models for taxonomic identification of a cryptic group of snails. The use of Siamese models specifically is a unique way to go approach such problems in taxonomy and I think it will be of general interest and application for other groups as well. But I do have a few major and minor recommendations before it should be accepted.

Major comments:

Although this is a unique approach to identifying cryptic snails, there are other papers that have been published recently that use CV methods for the same purpose and taxonomic group (molluscs). These should be discussed in the intro and/or discussion as appropriate because they have the same goals but use a different approach. The discussion in general is very lacking in citations (only 2!). For a paper like this, I would expect many more. How else is the reader supposed to put the results of your paper into the wider literature context if you have not cited anything in the discussion? So at the very least, you need to have a discussion around important papers that were published in this field (and taxonomic group) using CV methods:

Hollister, J.D., Paz-García, D.A., Beas-Luna, R., Horton, T., Cai, X. and Fenberg, P.B., 2025. Genes, shells, and AI: using computer vision to detect cryptic morphological divergence between genetically distinct populations of limpets. Scientific Reports.

Hollister, J.D., Cai, X., Horton, T., Price, B.W., Zarzyczny, K.M. and Fenberg, P.B., 2023. Using computer vision to identify limpets from their shells: a case study using four species from the Baja California peninsula. Frontiers in Marine Science, 10, p.1167818.

Line 70: there is an important paper to cite here as well by the same author as above that covers this topic:

Hollister, J.D., Martin, G., Cai, X., Horton, T., Powell, O., Sterling, M., Turnbull, G., Price, B.W. and Fenberg, P.B., 2025. A computer vision method for finding mislabelled specimens within natural history collections. Ecology and Evolution, 15(7), p.e71648.

While the methods are generally well described in terms of the model, there are some aspects of the dataset that are not clear. For example, the reader has to find their way to the Zenodo dataset to know what shell measurements are used for each specimen. All you say is that you used “shell measurements”. But this is very vague and does not tell the reader much. What shell measurements were used and why? Is there a big overlap in shell measurements between species and individuals? Molluscs have a large range in size and shape, so it’s important to give more context. Perhaps a table or SI table would be helpful with this information.

How did you train species that only have 5 specimens? Is that not too low of a sample size to accurately use them for training purposes? Seems like you need more justification on how accurate the models are for species where you have low samples sizes.

Line 416: why would you ever use a specimen in a test dataset if they are also in the training data? The whole point of the training data is so you can test it using un-seen data. This seems like a major flaw but I might be missing something. Related to this, you do not mention anywhere how many (or proportion) specimens are in the training, validation, and test datasets per species. Unless I am missing something fundamental here, I think this is a key piece of information that is not present in the study.

Minor comments:

Line 162: what does “drop substantially” mean in terms of statistics?

Line 169: how many “countries” are there in the dataset?

It’s hard to tell if you only have images of specimens or if you have the specimens themselves. So a “specimen” is just an image, not an actual physical specimen?

Are all images taken at the same angle, distance and orientation (e.g. aperture side up?) if so state this.

Line 35, add some average performance scores here (F1 scores?)

Lines 74-88: well written, but you are missing some recent studies that have looked at these issues you raise here, please cite papers by Hollister et al. They use CV to identify cryptic marine snails (limpets), both between and within species.

Line 116: cite Hollister et al. 2025, where they look at cryptic divergence across space within species using CV methods.

Line 162: what does “drop substantially” mean in terms of statistics?

Line 169: how many “countries” are there in the dataset?

It’s hard to tell if you only have images of specimens or if you have the specimens themselves. So a “specimen” is just an image, not an actual physical specimen?

Are all images taken at the same angle, distance and orientation (e.g. aperture side up?) if so state this.

Line 327: who is Adam?

Fig 5. The x-axis labels do not quite match with what is written in the caption or in the text. Include the “genetic data” at least on the appropriate area so we know which one you’re referring to. What is exactly the “test set performance” metric…is it an F1 score or similar?

There is a A LOT of hard to navigate jargon in lines 371-380. Please write this is plain language if possible, or explain a bit more.

What is the justification for the “top 3 accuracy” box plots in figs 5 and 6? What are the “top 3”, this is not explained in the paper.

Line 426-429: so there is a lot of intraspecific variability, did you confirm the correct species using genetic data for all specimens?

Figs 10 and 11 are very hard to interpret. Please re-do it so it;’s more visually appealing.

The discussion is very poorly cited (only 2 citations!). Even for a methods paper like this one, that is very very few for a discussion. How are we to put your study into a wider context with the literature with so few references to similar studies? Again, you should cite similar papers in the field, e.g. Hollister et al.

**********

**Have the authors made all data and (if applicable) computational code underlying the findings in their manuscript fully available?**

The PLOS Data policy requires authors to make all data and code underlying the findings described in their manuscript fully available without restriction, with rare exception (please refer to the Data Availability Statement in the manuscript PDF file). The data and code should be provided as part of the manuscript or its supporting information, or deposited to a public repository. For example, in addition to summary statistics, the data points behind means, medians and variance measures should be available. If there are restrictions on publicly sharing data or code —e.g. participant privacy or use of data from a third party—those must be specified.

Reviewer #1: Yes

Reviewer #2: Yes

Reviewer #3: Yes

**********

PLOS authors have the option to publish the peer review history of their article (what does this mean?). If published, this will include your full peer review and any attached files.

If you choose “no”, your identity will remain anonymous but your review may still be made public.

**Do you want your identity to be public for this peer review?**  For information about this choice, including consent withdrawal, please see our Privacy Policy.

Reviewer #1: No

Reviewer #2: **Yes:** David Moi

Reviewer #3: No

[NOTE: If reviewer comments were submitted as an attachment file, they will be attached to this email and accessible via the submission site. Please log into your account, locate the manuscript record, and check for the action link "View Attachments". If this link does not appear, there are no attachment files.]

**Figure resubmission:**

While revising your submission, we strongly recommend that you use PLOS’s NAAS tool (https://ngplosjournals.pagemajik.ai/artanalysis) to test your figure files. NAAS can convert your figure files to the TIFF file type and meet basic requirements (such as print size, resolution), or provide you with a report on issues that do not meet our requirements and that NAAS cannot fix.

After uploading your figures to PLOS’s NAAS tool - https://ngplosjournals.pagemajik.ai/artanalysis, NAAS will process the files provided and display the results in the "Uploaded Files" section of the page as the processing is complete. If the uploaded figures meet our requirements (or NAAS is able to fix the files to meet our requirements), the figure will be marked as "fixed" above. If NAAS is unable to fix the files, a red "failed" label will appear above. When NAAS has confirmed that the figure files meet our requirements, please download the file via the download option, and include these NAAS processed figure files when submitting your revised manuscript.

**Reproducibility:**

To enhance the reproducibility of your results, we recommend that authors of applicable studies deposit laboratory protocols in protocols.io, where a protocol can be assigned its own identifier (DOI) such that it can be cited independently in the future. Additionally, PLOS ONE offers an option to publish peer-reviewed clinical study protocols. Read more information on sharing protocols at https://plos.org/protocols?utm_medium=editorial-email&utm_source=authorletters&utm_campaign=protocols

# Author comment: Author response to Decision Letter 1

**DOI:** 10.1371/journal.pcbi.1014733.r002

23 Jul 2026

Attachment Submitted filename: Response to Reviewers.docx

# Peer review: Decision Letter 1

**DOI:** 10.1371/journal.pcbi.1014733.r003

**Authors:** Pedro Mendes, Miguel Francisco de Almeida Pereira de Rocha

17 Aug 2026

Dear Mr. Vetter,

We are pleased to inform you that your manuscript 'Speeding up taxonomy in the digital age: A deep learning approach for identifying cryptic freshwater snails' has been provisionally accepted for publication in PLOS Computational Biology.

Before your manuscript can be formally accepted you will need to complete some formatting changes, which you will receive in a follow up email. A member of our team will be in touch with a set of requests.

Please note that your manuscript will not be scheduled for publication until you have made the required changes, so a swift response is appreciated.

IMPORTANT: The editorial review process is now complete. PLOS will only permit corrections to spelling, formatting or significant scientific errors from this point onwards. Requests for major changes, or any which affect the scientific understanding of your work, will cause delays to the publication date of your manuscript.

Should you, your institution's press office or the journal office choose to press release your paper, you will automatically be opted out of early publication. We ask that you notify us now if you or your institution is planning to press release the article. All press must be co-ordinated with PLOS.

Thank you again for supporting Open Access publishing; we are looking forward to publishing your work in PLOS Computational Biology.

Best regards,

Miguel Francisco de Almeida Pereira de Rocha

Academic Editor

PLOS Computational Biology

Pedro Mendes

Section Editor

PLOS Computational Biology

***********************************************************

Reviewer's Responses to Questions

**Comments to the Authors:**

**Please note here if the review is uploaded as an attachment.**

Reviewer #1: The authors have done a good job in revising the manuscript. All issues have been properly addressed. I have no additional comments.

Reviewer #2: Thank you for addressing my comments. The plots showing attention on the image are interesting and show what features are salient in the classification. Any small doubts I had about the network 'cheating' in order to classify specimens have been dealt with. The addition of unsupervised training was appropriate and has improved results. I think the manuscript is ready to be published.

**********

**Have the authors made all data and (if applicable) computational code underlying the findings in their manuscript fully available?**

The PLOS Data policy requires authors to make all data and code underlying the findings described in their manuscript fully available without restriction, with rare exception (please refer to the Data Availability Statement in the manuscript PDF file). The data and code should be provided as part of the manuscript or its supporting information, or deposited to a public repository. For example, in addition to summary statistics, the data points behind means, medians and variance measures should be available. If there are restrictions on publicly sharing data or code —e.g. participant privacy or use of data from a third party—those must be specified.

Reviewer #1: Yes

Reviewer #2: Yes

**********

PLOS authors have the option to publish the peer review history of their article (what does this mean?). If published, this will include your full peer review and any attached files.

If you choose “no”, your identity will remain anonymous but your review may still be made public.

**Do you want your identity to be public for this peer review?**  For information about this choice, including consent withdrawal, please see our Privacy Policy.

Reviewer #1: **Yes:** Lai Wei and Qiaoxing Liang

Reviewer #2: No

# Editor report: Acceptance letter

**DOI:** 10.1371/journal.pcbi.1014733.r004

**Authors:** Pedro Mendes, Miguel Francisco de Almeida Pereira de Rocha

PCOMPBIOL-D-26-00450R1

Speeding up taxonomy in the digital age: A deep learning approach for identifying cryptic freshwater snails

Dear Dr Vetter,

I am pleased to inform you that your manuscript has been formally accepted for publication in PLOS Computational Biology. Your manuscript is now with our production department and you will be notified of the publication date in due course.

The corresponding author will soon be receiving a typeset proof for review, to ensure errors have not been introduced during production. Please review the PDF proof of your manuscript carefully, as this is the last chance to correct any errors. Please note that major changes, or those which affect the scientific understanding of the work, will likely cause delays to the publication date of your manuscript.

Soon after your final files are uploaded, unless you have opted out, the early version of your manuscript will be published online. The date of the early version will be your article's publication date. The final article will be published to the same URL, and all versions of the paper will be accessible to readers.

For Research, Software, and Methods articles, you will receive an invoice from PLOS for your publication fee after your manuscript has reached the completed accept phase. If you receive an email requesting payment before acceptance or for any other service, this may be a phishing scheme. Learn how to identify phishing emails and protect your accounts at https://explore.plos.org/phishing.

Thank you again for supporting PLOS Computational Biology and open-access publishing. We are looking forward to publishing your work!

With kind regards,

Sharmila Kamatchi

PLOS Computational Biology | Carlyle House, Carlyle Road, Cambridge CB4 3DN | United Kingdom ploscompbiol@plos.org | Phone +44 (0) 1223-442824 | ploscompbiol.org | @PLOSCompBiol
